# Epitaxial Fe Coating on Single- and Few-Layer Mo_2_C MXene as Highly Efficient Catalyst for Ambient Electrochemical Ammonia Synthesis

**DOI:** 10.1021/acsaem.5c02197

**Published:** 2025-09-16

**Authors:** Sabine Eliane Midré, Liang Tian, Hermenegildo García, Sara Goberna-Ferrón, Ana Primo

**Affiliations:** Instituto de Tecnología Química, Consejo Superior de Investigaciones Científicas-Universitat Politècnica de Valencia, Universitat Politècnica de Valencia, Av. De los Naranjos s/n, 46022 Valencia, Spain

**Keywords:** electrocatalytic nitrogen reduction reaction, MXene as electrocatalyst, 2D materials, epitaxial iron on Mo_2_C, operando ATR-SEIRAS

## Abstract

Iron-intercalated Mo_2_C MXene (Fe/Mo_2_C) is presented as a robust, earth-abundant electrocatalyst for ambient ammonia synthesis. An in situ HF protocol converts Mo_2_Ga_2_C MAX into few-layer Mo_2_C terminated with =O/–F groups; subsequent wet impregnation followed by mild reduction deposits 7 wt % Fe(0) as an epitaxial coating that lines the internal van der Waals galleries and simultaneously blankets the external basal planes of the Mo_2_C MXene. Aberration-corrected STEM, FFT/inverse-FFT analysis, and STEM-EDX depth profiling verify lamellar Fe domains that connect neighboring Mo_2_C sheets while preserving crystallinity. This hybrid architecture halves the charge-transfer resistance (EIS) and provides a high density of Fe sites that preferentially adsorb N_2_ over protons. In neutral 0.1 M Na_2_SO_4_, the optimized material achieves a Faradaic efficiency of 28.8% and an NH_3_ yield of 19.1 μmol h^–1^ mg_Fe_^–1^ at 0.25 V vs RHE, matching or surpassing noble-metal benchmarks under ambient conditions. Operando ATR-SEIRAS detects N–N and −NH*_x_* vibrations consistent with an associative pathway, and ^15^N_2_ labeling confirms the nitrogen source. The catalyst maintains stable performance for 10 h with no detectable Fe leaching. Thus, the combination of the activity of Fe overlayers with the conductive, mechanically resilient Mo_2_C framework renders a material with remarkable electrocatalytic activity for green-ammonia production.

## Graphical abstract

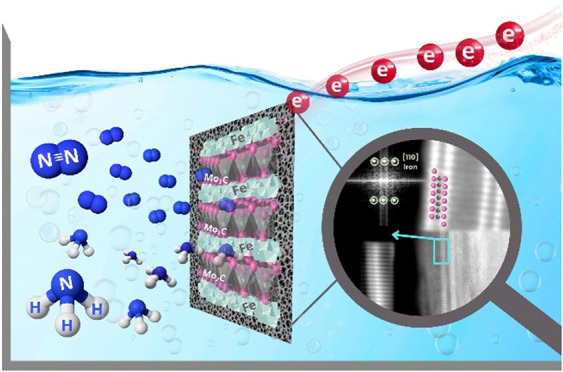

## Introduction

Ammonia underpins both food security and the emerging green-energy economy. With an annual production close to 230 million tonnes,^1^ about 80% of which is converted into fertilizers that nourish roughly half of the world’s population,^1^ the century-old Haber–Bosch process consumes 1–2% of global energy and emits up to 3% of anthropogenic CO_2_.^2^ Due to its high H percentage in weight, ammonia is also gaining traction as a carbon-free hydrogen vector, with a high volumetric energy density (12.7 MJ L^–1^), being easy to liquify at −33 °C at ambient pressure or at room temperature under 8 bar, ammonia surpasses the volumetric energy density of liquid hydrogen, enabling a convenient storage and direct utilization in high-temperature fuel cells. Formation of “green” ammonia at ambient conditions, however, requires electrocatalysts that can activate the inert N≡N bond while suppressing the parasitic hydrogen evolution reaction (HER).^3^

Two-dimensional MXenes, first exfoliated from MAX phases by Gogotsi, Barsoum, and co-workers in 2011,^4^ offer metallic electrical conductivity, abundant surface terminations, and a lamellar architecture that can support catalytically active motifs within ångström-wide galleries. Mo_2_C MXene is particularly attractive because its carbide framework has some resemblance to the electron-rich environment of iron–molybdenum cofactors in natural nitrogenases, yet its direct exposure to electrolyte renders surface-deposited metals prone to aggregation and leaching. In nature and in the industrial Haber–Bosch loop alike, iron is the ubiquitous center for N_2_ fixation, motivating the design of earth-abundant Fe catalysts that emulate the MoFe enzymatic site able to operate under mild conditions.^5,6^ Biomimetic rationale underpins our catalyst design: in the natural enzyme, the FeMo-cofactor is a [Mo_7_Fe_9_S_9_C] cluster whose tightly packed Fe atoms and interstitial C create an electron-rich pocket that polarizes and cleaves the N≡N bond at room temperature.^8,9^ Translating this motif to a solid material, zerovalent Fe atoms were intercalated between the conductive Mo_2_C sheets of a MXene so that the Fe atoms act as metallic “bridges,” while the carbide framework supplies the Mo–C environment that shuttles electrons to the adsorbed N_2_, thus emulating both the composition and the three-dimensional confinement of the enzymatic active site.

Herein, we report a wet impregnation/reduction route in which HF-etched Mo_2_C MXene spontaneously intercalates zerovalent Fe atoms between its layers, creating metallic Fe bridges that (i) form an epitaxial overcoat of single-layer Mo_2_C, (ii) mechanically stitch adjacent Mo_2_C sheets in few-layer particles by occupying the intergallery space, (iii) open vertical electron-transport pathways that lower the charge-transfer resistance, and (iv) generate a confined, electron-rich microenvironment that favors N_2_ adsorption over protons. Compared to other alternatives like electrochemical metal deposition,^7^ the present method is considerably simpler since it basically consists of impregnation of the solid in suspension. Optimizing the loading to 7 wt % Fe yields a homogeneous interlayer distribution with no detectable nanoparticles and drives the Faradaic efficiency to 28.78% with an NH_3_ yield of 19.14 μmol h^–1^ mg_Fe_^–1^ (1.34 μmol h^–1^ mg_cat_^–1^) at −0.25 V vs RHE in neutral electrolyte, figures that rival noble-metal benchmarks while retaining structural integrity over extended operation. The use of MXene as electrocatalysts for electrochemical nitrogen reduction reaction has been recently reviewed and the activity data achieved with Fe supported Mo_2_C are among the best efficiencies reported so far.^8–10^ By coupling the activity of iron with the conductive scaffold of Mo_2_C, the interlayer Fe strategy establishes a robust, scalable platform for the sustainable electrosynthesis of ammonia.

## Experimental Section

### Materials

Commercially available Mo_2_Ga_2_C from Nanochemazone was used as a precursor. HCl, NH_4_F, and iron acetate for the delamination and impregnation were supplied by Sigma-Aldrich.

### Synthesis of Mo_2_C MXene

Mo_2_C MXene was synthesized using the in situ HF etching method, chosen over direct HF handling due to enhanced safety. To generate in situ HF, 1.5 g of NH_4_F was dissolved in 9 mL of ultrapure Milli-Q water and mixed with 15 mL of HCl. Subsequently, 0.5 g of Mo_2_Ga_2_C was added to the solution. The resulting suspension was heated to 55 °C and stirred continuously for 96 h. After the reaction, Mo_2_C was filtered, thoroughly washed with ultrapure water, and dried.

### Synthesis of Fe/Mo_2_C

A total of 100 mg of Mo_2_C was dispersed in 10 mL of Milli-Q water, shaken, and then sonicated in a water bath for 10 min. Separately, iron acetate was dissolved in 10 mL of Milli-Q water and stirred until fully dissolved. Varying amounts of iron acetate were used to achieve different concentrations, as detailed in Table S1. The Mo_2_C MXene dispersion was then added dropwise to the iron acetate solution. The resulting suspension was heated to 80 °C with vigorous stirring for 20 h. Following the reaction, the material was sonicated, filtered, and dried at 60 °C for 2 h.

#### Physicochemical Characterization

Inductively coupled plasma-optical emission spectroscopy (ICP-OES) elemental analyses were performed on a PerkinElmer Avio 560 Max instrument after dissolving the samples with a mixture of HF/HNO_3_/HCl.

X-ray photoelectron spectroscopy (XPS) analyses were performed using a SPECS spectrometer equipped with a Phoibos 150 MCD-9 detector using nonmonochromatic Mg Kα radiation (50 W, 1253.6 eV) and a multichannel detector. The C 1 s signal of carbon at 284.8 eV was used as a reference for calibration. For depth profiling, monatomic Ar+ ion bombardment with an energy of 5 kV was employed for the removal of the outermost external layers. The pressure was 1 × 10^–7^ mbar, and sputtering was conducted for 20 min. XPS data were processed by using Casa XPS software, applying the Shirley function for background correction.

X-ray diffraction (XRD) patterns were acquired in Bragg–Brentano geometry using a PANalytical CUBIX diffractometer equipped with a PANalytical X’Celerator detector. X-ray radiation of Cu Kα (λ_1_ = 1.5406 Å, λ_2_ = 1.5444 Å, *I*_2_/*I*_1_ = 0.5) was used, with a tube voltage and an intensity of 45 kV and 40 mA, respectively. The length of the arm of the goniometer was 200 mm, and a slit of variable divergence with an irradiated sample area of 3 mm was used. The measurement range was from 2.0 to 90.0° (2θ), with a step of 0.020° (2θ) and a counting time of 17 s per step. The measurement was carried out at 298 K, rotating the sample at 0.5 revolutions/s.

FESEM images were acquired by using a ZEISS GeminiSEM 500 electron microscope having an EDX detector. The samples were deposited directly on a sticky carbon support attached to the sample holder.

Structural and morphological analyses were conducted by using high-angle annular dark-field scanning transmission electron microscopy (HAADF-STEM) and high-resolution TEM (HR-TEM). Samples were dispersed in ethanol with ultrasonic treatment for 2 min and then deposited onto a 400-mesh copper microgrid. Imaging and energy-dispersive X-ray spectroscopy (EDS) were performed on a Titan G2 (60-300) aberration-corrected transmission electron microscope operating at an acceleration voltage of 300 kV. Transmission electron microscopy (TEM) images of the Fe/Mo2C-1 and Fe/Mo2C-3 samples were taken in a Bruker electron microscope under 200 kV as the accelerating voltage.

The thickness of the pristine MXene was determined by atomic force microscopy (AFM) analysis with a Multimode Nanoscope 3 A instrument.

#### Electrochemical Characterization

Nickel foam, with a thickness of 0.9 mm, was used from Goodfellow. Aquivion D72-25BS from Sigma-Aldrich was used as the fluoropolymer binder. UV–vis spectra in the range of 200–800 nm were recorded using a Cary 300 Scan UV–visible spectrophotometer. NH_3_ concentration was measured with the help of a Spectroquant Ammonium Test with a photometric method from Sigma-Aldrich.

Electrocatalyst inks were prepared by dispersing 1 mg of catalyst with 120 μL of Milli-Q water and 120 μL of 2-propanol for 15 min in a sonicator bath. Then, 1 μL of Aquivion Binder was added, and the mixture was sonicated again. In the meantime, the nickel foam electrodes were cut into 5 × 15 mm^2^ rectangles. Before the ink was drop-cast on the electrodes, they were previously washed, dried, and heated under an IR lamp. The electrodes were cleaned by a multistep washing process. First, they were immersed in acetone and sonicated for 15 min. After being rinsed with ethanol, they were sonicated again, followed by rinsing with Milli-Q water. Finally, the electrodes underwent an additional sonication step in Milli-Q water and a final rinse with Milli-Q water. Once the ink was prepared, it was drop-cast onto the Ni foam, with 60 μL deposited on each side of the Ni foam. Care was taken to cover only the lower two-thirds of the electrode surface. This assures us to have a well-dispersed loading of 0.5 mg cm^–2^. The as-prepared electrodes were dried overnight in a 60 °C oven before usage.

Electrochemical tests were performed using a Gamry Interface 500 E Potentiostat/Galvanostat. *iR* compensation for the internal resistance was not applied. An H-cell three-electrode setup was used, with one compartment holding the Pt coil counter electrode. The other compartment held the reference electrode (Ag/AgCl for pH from 1 to 9 and Hg/HgO for pH 13). Both compartments were separated by an activated Nafion membrane. Additionally, ammonia traps were connected upstream to remove any residual ammonia from the nitrogen bottle to enter the H-cell system and downstream to detect and quantify any ammonia that escapes the system. After every 2 h of electrochemical measurement, the electrolytes from each compartment of the H-cell and the trap were analyzed for their ammonia content. Potentials are reported against the reversible hydrogen electrode (RHE) using the following formula: *E*_RHE_ = *E*_ref_ + *E*_ref_^0^ 0.0592 × pH. Table S2 shows the used electrolytes and buffers and their respective pHs.

Electrochemical impedance spectroscopy (EIS) was also performed with the Gamry Interface 500 E Potentiostat. The initial frequency was 1 MHz, and the final frequency was about 1 mHz. The amplitude of the AC voltage was 5 mV. The cyclic voltammetries (CV) and linear sweep voltammetries (LSV) were made from a potential of −0.15 V vs RHE to 1.15 V vs RHE with a scan rate of 50 mV s^–1^.

Before each measurement, a CV was executed to get rid of potential oxidative species on the surface of the electrode, for as many cycles as needed to stabilize the signal. For each material, an LSV was executed in both argon- and nitrogen-saturated electrolytes to determine the overpotential change of the catalyst due to nitrogen reduction. To measure the performance of the catalyst for the eNRR, chronoamperometry (CA) for 2 h at different potentials was performed. Given the known quantities of catalyst used, the volume of the electrolytes in the H-cell and the trap, and the duration of the reaction, the yield per hour can be calculated. The ammonia concentration is determined by employing the ammonia test kit from Sigma-Aldrich using the indophenol blue method. The intensity of the blue color, measured with a spectrophotometer, is directly proportional to the ammonia concentration. Calibration is necessary to correlate the color intensity with ammonia concentration. The calibration results and the respective *R*-square factors are shown in Figure S1. The samples were measured in the spectrophotometer exactly 20 min after the test kit was applied.

To measure the performance of an electrocatalyst for the eNRR, two important values are studied: the faradic efficiency (FE) and ammonia yield. The FE measures how much of the invested energy is converted into the desired product, in our case ammonia.^7^ Yield refers to the amount of the desired product produced. They have a strong correlation since the FE is calculated with the yield. To our knowledge, no literature reports a FE for eNRR in aqueous electrolytes exceeding 50%. This limitation arises from the occurrence of competitive hydrogen evolution reaction (HER) in aqueous media, which becomes predominant at higher potentials.^7,8^ The HER competes with the eNRR, significantly reducing the efficiency of nitrogen reduction due to the preferential formation of hydrogen gas over ammonia at elevated potentials.

The yield and FE were calculated using the following equations:

$$yield=\frac{\sum c^{\;}\times V}{M}$$

$$FE={\frac{{yield}^{\;}\times e^{\;}\times F}{Q}}^{\;}\times 100\%$$

where *c* is the concentration obtained through our measurement with the spectrophotometer and the calibration, *V* is the volume of the electrolyte in the compartments in the H-cell, and *M* is the product molar mass, in our case ammonia. To calculate the FE, we needed the previously calculated yield. The charge transferred per molecule “*e*” corresponds in our case to 3 electrons. F is the Faraday constant, and *Q* is the total charge transferred during the reaction, obtained by integrating the current during the CA. No correction for the internal ohmic losses of the H-cell was considered in these calculations.

Surface-enhanced infrared absorption spectroscopy (SEIRAS) in the attenuated total reflection (ATR) configuration was employed to investigate the electrochemical interface. A Thermo Scientific Nicolet iS50 FTIR spectrometer equipped with a liquid-nitrogen-cooled mercury cadmium telluride (MCT) detector was used for the electrochemical ATR-SEIRAS measurements. The ATR substrate was a trapezoidal ZnSe prism. A 5 nm titanium adhesion layer was first deposited onto the prism via electron beam evaporation (Temescal FC-2000) at a rate of 0.1 Å s^–1^ to enhance the mechanical stability of the subsequent metal film. Subsequently, an approximately 30 nm thick gold layer was deposited at the same rate to serve as the plasmonic substrate. The catalyst ink was prepared by ultrasonically dispersing 5 mg of catalyst in a mixed solvent of deionized water and isopropyl alcohol (volume ratio 1:4) for 2 h. Then, 40 μL of the ink was drop-cast onto a gold-coated prism and dried in air to form the working electrode. A platinum wire was used as the counter electrode, and a Ag/AgCl (3 M KCl) reference electrode was positioned close to the working electrode via a Luggin capillary. The electrolyte was N_2_-saturated 0.1 M Na_2_SO_4_. The spectra were collected by using a CHI 760e electrochemical workstation to control the potential. A constant potential of 0 V vs RHE was applied and maintained for 30 min before spectrum acquisition (800 scans averaged). Spectra were subsequently recorded at 0.1 V potential intervals. Unless otherwise stated, the spectral resolution was set to 8 cm^–1^ for all measurements.

Isotopic labeling experiments were carried out following the standard experimental procedure, but bubbling ^15^N_2_ into the system, and the electrochemical test was conducted for 2 h at a potential of −0.25 vs RHE. Following the experiment, the electrolyte was collected from both compartments of the H-cell as well as from the ammonia trap and analyzed using UV–vis spectroscopy. The electrolyte from the working compartment, which exhibited the highest ammonia concentration, was subsequently acidified with HCl to adjust the pH to approximately a value of 2. A volume of approximately 200 μL of this acidified solution was transferred into an NMR tube, followed by the addition of approximately 400 μL of perdeuterated dimethyl sulfoxide (DMSO-*d*_6_). The tube was shaken to ensure thorough mixing of the components. Proton nuclear magnetic resonance (^1^H NMR) spectra were then recorded using a Bruker AV400 (400 MHz) spectrometer, with signal averaging performed over 256 scans.

## Results and Discussion

Selective removal of Ga from the parent MAX phase Mo_2_Ga_2_C was carried out by in situ HF generation that diminishes the hazards associated with the manipulation of concentrated HF.^11^

A mixture of HCl, NH_4_F, and deionized water (Scheme 1) generates in situ stoichiometric HF amounts according to the following equation:

$${\mathrm{NH}}_{4}F+\mathrm{HCl}\to {\mathrm{NH}}_{4}\mathrm{Cl}+\mathrm{HF}$$

**Scheme 1:**
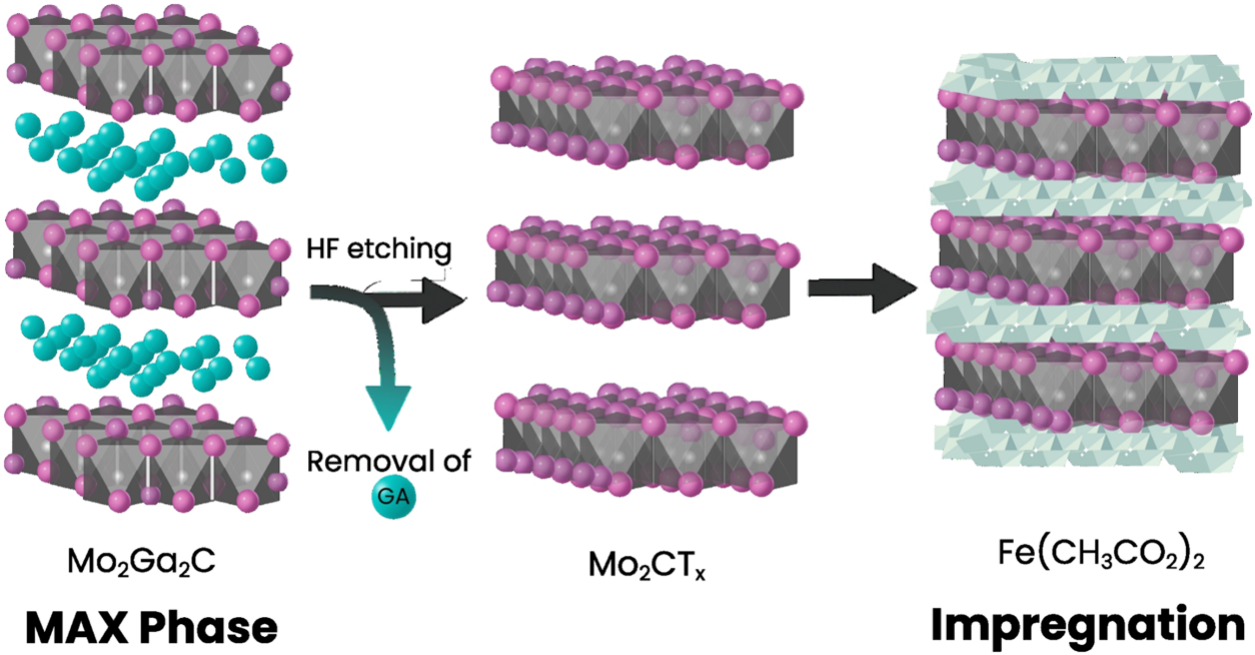
Pictorial Illustration of the Synthesis Procedure Followed to Prepare Fe-Intercalated Mo_2_C MXene^a^

a (i) In situ HF generated from an HCl/NH_4_F/H_2_O mixture selectively etches Ga layers from the Mo_2_Ga_2_C MAX precursor, yielding delaminated Mo_2_C sheets terminated with =O groups. (ii) Open interlayer galleries are impregnated overnight with an aqueous Fe^2+^ precursor; subsequent mild thermal reduction converts the incorporated ions to zerovalent Fe, forming conformal metallic Fe bridges that stitch adjacent MXene layers while preserving the two-dimensional carbide lattice.

After etching, surface termination and partial delamination occur in a single step. The overall reactions may be written as

$${\mathrm{Mo}}_{2}{\mathrm{Ga}}_{2}C+6\mathrm{HF}\to 2{\mathrm{GaF}}_{3}+{\mathrm{Mo}}_{2}C+3H_{2}↑$$

$${\mathrm{Mo}}_{2}C+H_{2}O\to {\mathrm{Mo}}_{2}\mathrm{CO}+H_{2}↑$$

$${\mathrm{Mo}}_{2}C+2\mathrm{HF}\to {\mathrm{Mo}}_{2}{\mathrm{CF}}_{2}+H_{2}↑$$

Worth noting is that the present Mo_2_C preparation procedure is adequate for a gram scale. Upscaling to larger amounts would require the recovery of fluorinated reagents and their reuse, since otherwise the process would have a large environmental impact and not meet economic feasibility.

The freshly produced MXene sheets therefore bear mostly =O and −F terminations that are known to enhance hydrophilicity and confer colloidal stability in water. The etching process and the concentration of the etchant strongly determine the surface terminations, functional groups, defect density, and interlayer spacing of the resulting MXene and its delamination. Experimental parameters, including etching time and etchant concentration, are critical for the uptake of heteroatoms, as in our case iron, and the stability of the metal anchoring in the MXene structure.^12^

There are several ways to introduce heteroatoms into the MXene crystal, each of which offers distinct advantages in tuning material properties. One of them is via molten salt etching with Lewis acids, of which the cations are transition metals. The salts act like oxidizing agents, exchanging the oxidized aluminum with the reduced metal.^13,14^ This method is able to penetrate within all of the layers of the MAX phase, turning it into the MXene. Delamination of molten salt-prepared MXene clay has been proven difficult due to the hydrophobicity of the resulting surface terminations, stacking the layers closely together, without the ability for intercalants to enter, and therefore reducing the availability of active sites.^15^ For this reason, other methods for heteroatom doping of MXenes post-etching were investigated. Single-atom doping strategies, such as atomic layer deposition, enable isolate transition metal atoms to anchor in on available surface sites.^16–20^ The drawback for this method is that the deposited atoms are only on top of the surface, potentially limiting intercalation within the MXene and lowering the chemically active surface area. The BET surface area of powder MXenes is typically very low, probably due to the inability of N_2_ gas to access the interlayer space of dry powder samples where strong van der Waals forces exist. Another method to produce heteroatoms on a substrate is wet impregnation, it enables a strong control on the amount of metal that is incorporated into the MXene structure by changing the synthesis conditions, like the temperature, time, and salt concentration of the impregnation solution.^12,21^ Since the solution is stirred vigorously, an intercalation of the metal salts between the layers of the MXene is facilitated, and a homogeneous distribution is favored. Due to its versatility and the simplicity of wet impregnation, it is one of the most popular methods for heterodoping Mxenes. While other carbon-based supports often need a reductive step to anchor the metal salt on the substrate surface,^22,23^ this additional step is not necessary with MXenes. This is due to MXenes and in particular Mo_2_C MXene being rich in surface defects.^24^

Iron was introduced by overnight impregnation of the etched MXene in aqueous Fe^2+^ solutions spanning 3 orders of magnitude in concentration (10^–3^–10^–1^ M). Inductively coupled plasma-optical emission spectroscopy (ICP-OES) revealed a linear uptake of Fe with respect to the precursor concentration (Table S1 and Figure S2), demonstrating that the open lamellar architecture provides accommodation sites without achieving saturation in the explored Fe loading range. A nominal loading of 7 wt % (Fe/Mo_2_C-3), corresponding to the inflection point in catalytic activity discussed below, was selected for detailed characterization. Scheme 1 provides a pictorial illustration of the synthetic route used to obtain Fe-intercalated Mo_2_C MXene from its Ga-containing MAX precursor.

Powder X-ray diffraction data of the parent MAX, the etched Mo_2_C MXene, and the Fe-intercalated sample are compared in Figure 1 and Table 1. After etching, the intense (002) reflection shifts from 2θ = 9.8 to 7.29°, consistent with an expanded *d*-spacing of 12.1 Å that signals the successful removal of Ga layers and insertion of surface terminations.

**Figure 1:**
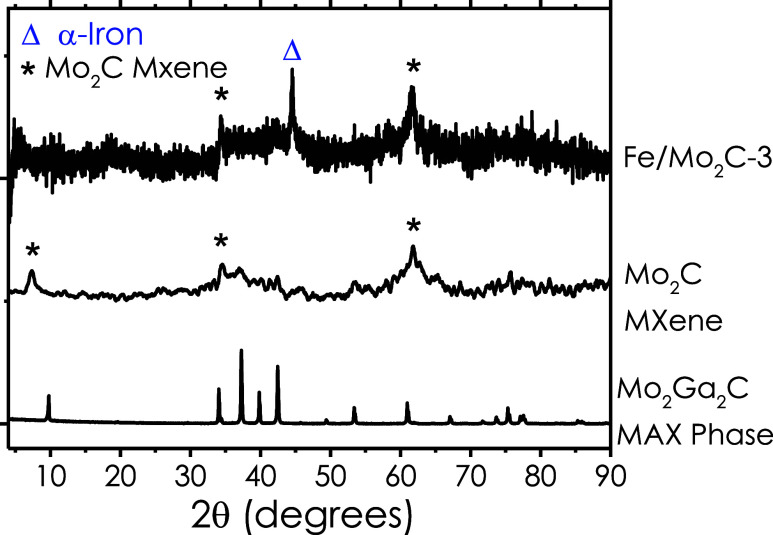
PXRD patterns of Fe/Mo_2_C-3, Mo_2_C MXene, and Mo_2_Ga_2_C MAX phase. The diffraction peaks marked with Δ correspond to α-Fe, indicating the presence of metallic iron in the FeMxene-3 sample. Peaks labeled with * are characteristic of Mo_2_C Mxene, confirming successful etching and transformation from the Mo_2_Ga_2_C MAX phase.

**Table 1:** XRD Peak Positions and Interatomic Distances of Pristine Mo_2_C and α-Fe with the Respective Miller Indices as Simulated with the Software Recipro

	MXene			Fe (α phase)		
*d* (Å)	12.1	2.6	1.5	2	1.4	1.2
angle (2θ)	7.29	34.5	61.5	44.6	65	82.3
Miller indices	(002)	(100)	(110)	(110)	(100)	(211)

Upon impregnation/reduction, two additional features emerge: (i) the (002) peak broadens and moves slightly to lower angle (8.2°), indicating further gallery expansion caused by iron atoms residing between Mo_2_C sheets; and (ii) weak reflections at 44.6 and 65° match the (110) and (100) planes of body-centered-cubic Fe(0), corroborating the presence of metallic iron rather than oxide or carbide derivatives. No peaks attributable to Fe_2_O_3_ or Fe_3_O_4_ are detected, confirming that the reduction step preserves the Fe zerovalent state, also inferred from XPS (see below). Collectively, the XRD data validate that the synthetic route yields a conformal overcoat of Fe on the sheets of Mo_2_C, and by filling the intergallery space of few-layer Mo_2_C particles, metallic iron stitches MXene sheets while the two-dimensional carbide lattice remains intact.

To obtain further information about the delamination achieved during the *in situ* etching step, atomic force microscopy (AFM) height mapping was performed on drop-cast dispersions of the as-produced MXene (Figure 2a). The topographic profiles disclose a heterogeneous population of flakes. A significant fraction consists of individual sheets whose apparent thickness averages (3.8 ± 0.4) nm, a value that matches a single Mo_2_C layer terminated on both sides by =O groups plus the adsorbed water layer typically retained under ambient AFM conditions. These monolayer domains, which extend laterally over several micrometers, testify to the efficiency of the in situ HF route in breaking the Mo–Ga bonds and liberating intact Mo_2_C lamellae.

**Figure 2:**
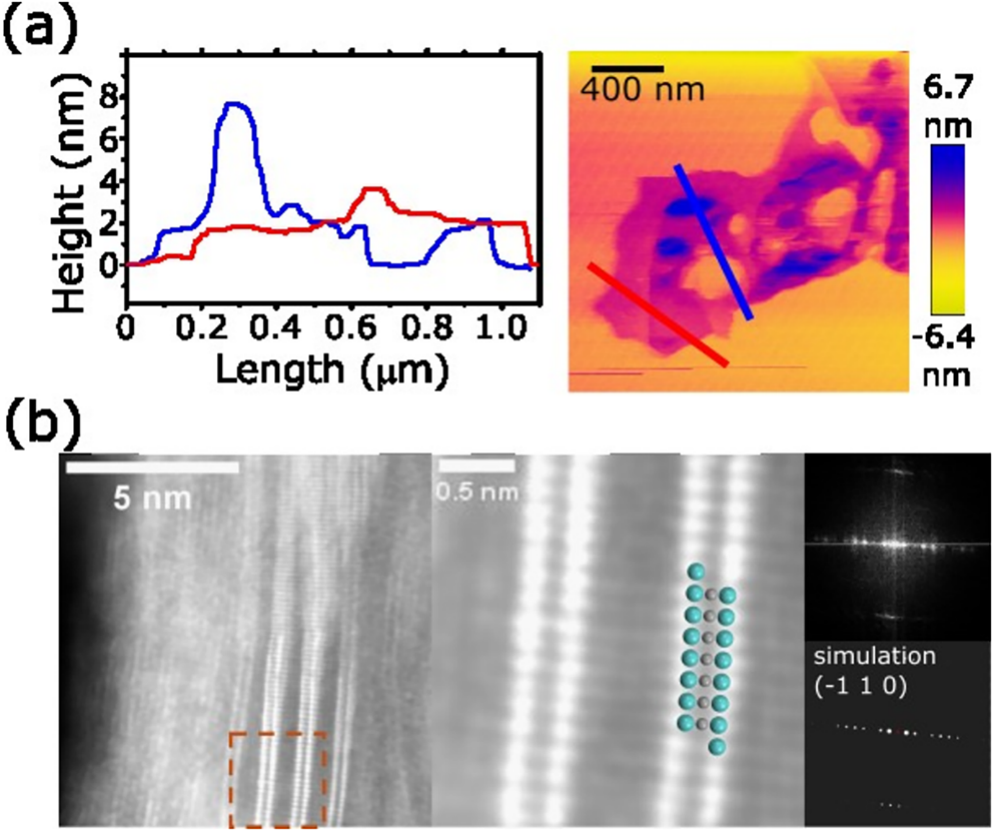
(a) Tapping-mode AFM height map of drop-cast dispersions of Mo_2_C and the corresponding height profiles. (b) Left: Cross-sectional aberration-corrected ADF-STEM of Fe/Mo2C-3. The orange box is the region enlarged in the center: view overlaid with the structural model of α-Mo_2_C along the (−1 1 0) zone axis; Mo (green) and carbide C (gray) (interlayer Fe atoms not resolved). Right, top: fast-Fourier transform (FFT) showing sharp reflections that index to the (−1 1 0) projection of α-Mo_2_C. Right, bottom: simulated diffraction pattern for the same orientation.

Interspersed among the monolayers, however, we detect platelet stacks with heights ranging from 5 to 7.7 nm, corresponding to two- to three-layer aggregates that have not undergone complete delamination. Their occurrence is most pronounced near the edges of drying droplets, suggesting that capillary forces during solvent evaporation promote the restacking of thinner flakes. The coexistence of monolayers and few-layer stacks is beneficial for subsequent Fe impregnation: single sheets maximize the external surface area and expose basal-plane terminations that trap Fe^2+^ ions, whereas lamellar regions provide confined galleries where the reduced Fe(0) can lodge as interlayer “bridges.” This hierarchical morphology therefore combines a high catalytic surface with robust scaffold domains, a feature that will become beneficial in the electrochemical performance.

High-resolution transmission electron microscopy (HR-TEM) and aberration-corrected scanning TEM (AC-STEM) were employed to obtain images and sub-ångström insights into the crystallographic registry and spatial disposition of Fe within the Mo_2_C MXene host. Whereas ensemble techniques such as XRD or XPS average over entire powders, lattice-resolved annular dark-field (ADF) images and their corresponding fast-Fourier transforms (FFTs) resolve individual galleries and discriminate, via atomic-number contrast, the heavier Mo columns from the lighter Fe species. This capability allows us to establish unequivocally whether iron resides as an epitaxial surface film, as lamellar interlayer bridges, or as isolated nanoparticles. The TEM micrographs that follow, therefore, provide a direct structural fingerprint of the dual iron population integrated into the two-dimensional carbide scaffold and lay the groundwork for correlating architecture with the electrocatalytic behavior. Figure 2b presents an aberration-corrected ADF-STEM overview of Fe/Mo_2_C-3. Continuous, straight lamellae run over several tens of nanometers with no tears or turbostratic waviness, confirming that the *in situ* HF etch liberates pristine Mo_2_C layers. The associated FFT indexes exclusively to α-Mo_2_C, demonstrating that the carbide lattice survives chemical treatment without amorphization.

A higher-magnification cross section (Figure 2b, left) resolves individual galleries; the mean interplanar spacing is 1.10 ± 0.03 nm, in excellent agreement with the *d*(002) value extracted from X-ray diffraction. Each gallery appears as two parallel rows of bright dots. Z-contrast simulations (Figure 2b, right) show that these dots correspond to Mo atomic columns viewed along the (−1 1 0) axis, where rows of Mo atoms alternate with rows of carbidic C atoms. FFTs taken from the same view reproduce the calculated diffraction pattern of the (−1 1 0) projection, corroborating the crystallographic assignment. Side-view TEM images collected from different flakes (Figure S3) yield the same 1.1 nm gallery distance, confirming that the spacing is homogeneous throughout the specimen.

Local variations in crystal orientation are expected for a polycrystalline, few-layered material; indeed, FFTs obtained a few nanometers away index to the (0 1 0) zone axis of Mo_2_C (Figure S4). In this projection, the Mo columns are aligned directly opposite one another across the interlayer, leading to the paired-dot motif seen experimentally. The coexistence of (−1 1 0) and (0 1 0) orientations therefore reflects random flake tilting rather than structural disorder.

Evidence of Fe incorporation is provided by the dual-domain ADF-STEM micrograph in Figure 3. The FFT extracted from the orange-boxed area (Figure 3a) displays a square array of reflections that index unambiguously to bcc-Fe viewed along the (1 1 0) axis. Because this signal is strongest at the outermost edge of the flake, it is assigned to an ultrathin Fe(0) overlayer that coats the external surface of the Mo_2_C stack in an epitaxial relationship with the carbide lattice. The concomitant presence of α-Mo_2_C spots in the same diffraction pattern confirms that the metal film is conformal rather than forming isolated nanoparticles.

**Figure 3:**
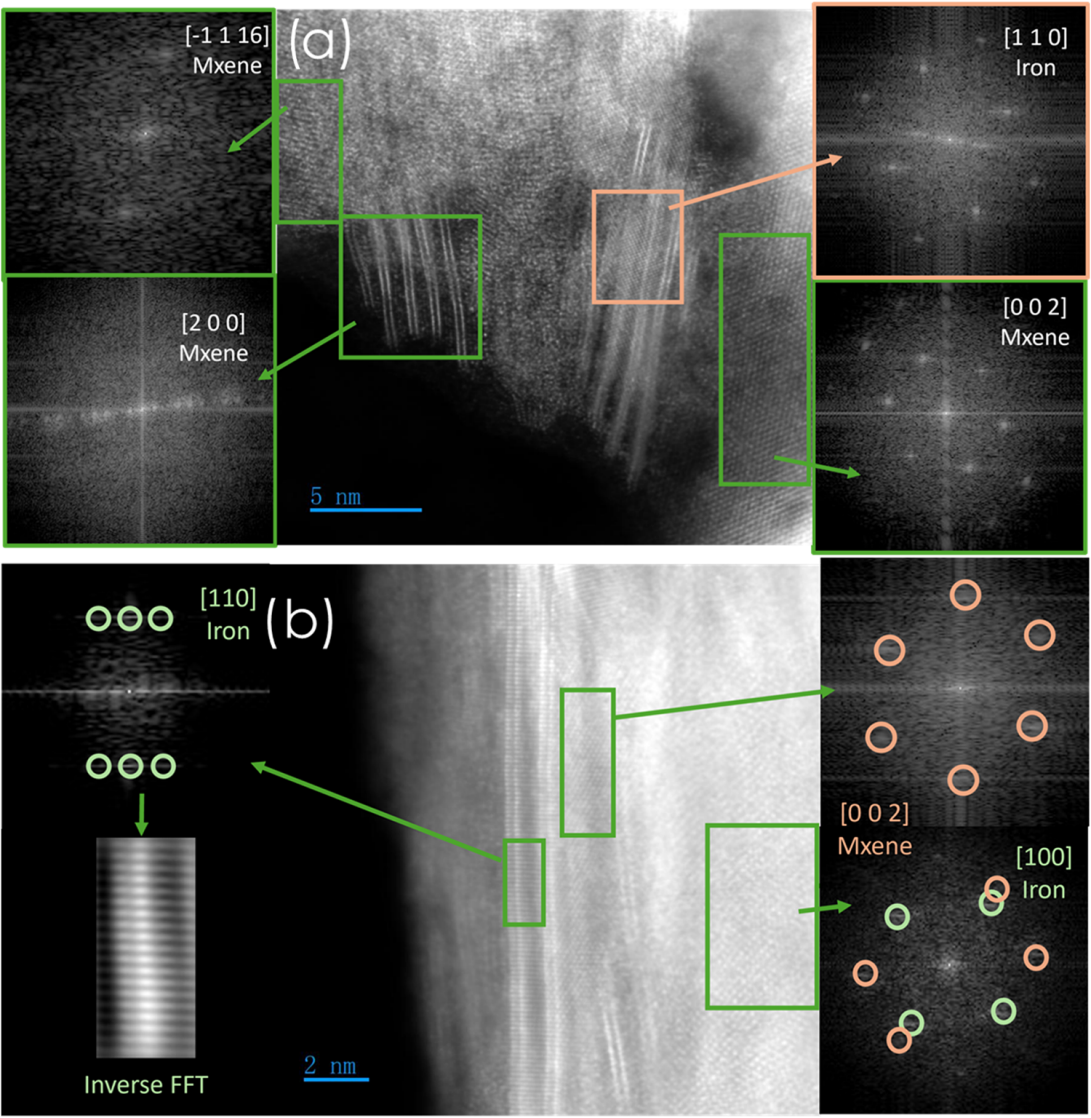
ADF-STEM cross sections of Fe-intercalated Mo_2_C MXene. (a) Low-magnification view with FFTs: green boxes give α-Mo_2_C zone axes, and orange FFT shows bcc-Fe (110) from a surface overlayer. (b) Atomic-resolution image; FFT reveals overlapping MXene (002) (orange) and bcc-Fe (100) (green) reflections. Inverse FFT of Fe spots (left) isolates 0.204 nm stripes, confirming lamellar Fe(0) bridges inside the galleries.

Additional insight is obtained by masking the Fe reflections and performing an inverse FFT (Figure 3b). The filtered image reveals a series of parallel stripes of intermediate contrast running between successive Mo rows, with an interplanar spacing of *d*_(110)_ ≈ 0.204 nm, which is the crystallographic fingerprint of metallic iron. These data firmly demonstrate that Fe(0) is not limited to a surface coating: a significant fraction nucleates inside the van der Waals galleries, adopting a lamellar, conformal morphology that *stitches* adjacent Mo_2_C sheets without disrupting the parent lattice. The combined FFT/inverse-FFT analysis therefore indisputably establishes a dual disposition of iron, external ultrathin film, plus interlayer bridges, both coherently integrated into the MXene scaffold, a configuration that appears to be optimal for a good electrical connectivity and catalytic site density in the electrochemical N_2_ to NH_3_ reaction. Elemental mapping by STEM-EDX further corroborates this conclusion (Figure S5). At low magnification, the annular dark-field image outlines an entire multilayer flake, while the superimposed Mo Kα (red) and Fe Kα (green) maps reveal that both elements are distributed uniformly across the full lateral dimension of the platelet and throughout its projected thickness; no Fe-depleted borders or Mo-rich/Fe-poor islands are discernible. At higher magnification (right panel of Figure S5), the same homogeneous overlap of Mo and Fe persists down to the few-nanometer scale, with the speckled texture arising from statistical noise rather than compositional segregation. The absence of discrete, Fe-only agglomerates, together with the FFT evidence of bcc-Fe reflections concentrated at the flake periphery, supports a model in which an ultrathin, conformal Fe(0) overlayer envelopes the Mo_2_C scaffold, while a complementary fraction of Fe intercalates between galleries as lamellar bridges. Such dual placement maximizes active-site density yet preserves the intrinsic conductivity and structural integrity of the MXene host.

Element-specific STEM-EDX line profiling (Figure 4a) quantifies the distribution of the constituent elements across a stitched region of the flake. Carbon (blue) and molybdenum (red) signals rise simultaneously upon crossing the particle edge and then plateau, consistent with the uniform stoichiometry of crystalline Mo_2_C. The Fe K-α counts (black) are negligible outside the flake, increase sharply at the same entry point, and display three periodic maxima that coincide with the projected gallery positions (shaded bands). The modulation of the Fe signal across the slab indicates that iron is strongly associated with the van der Waals galleries, supporting the presence of interlayer Fe bridges inferred from HR-STEM and XPS depth profiling.

**Figure 4:**
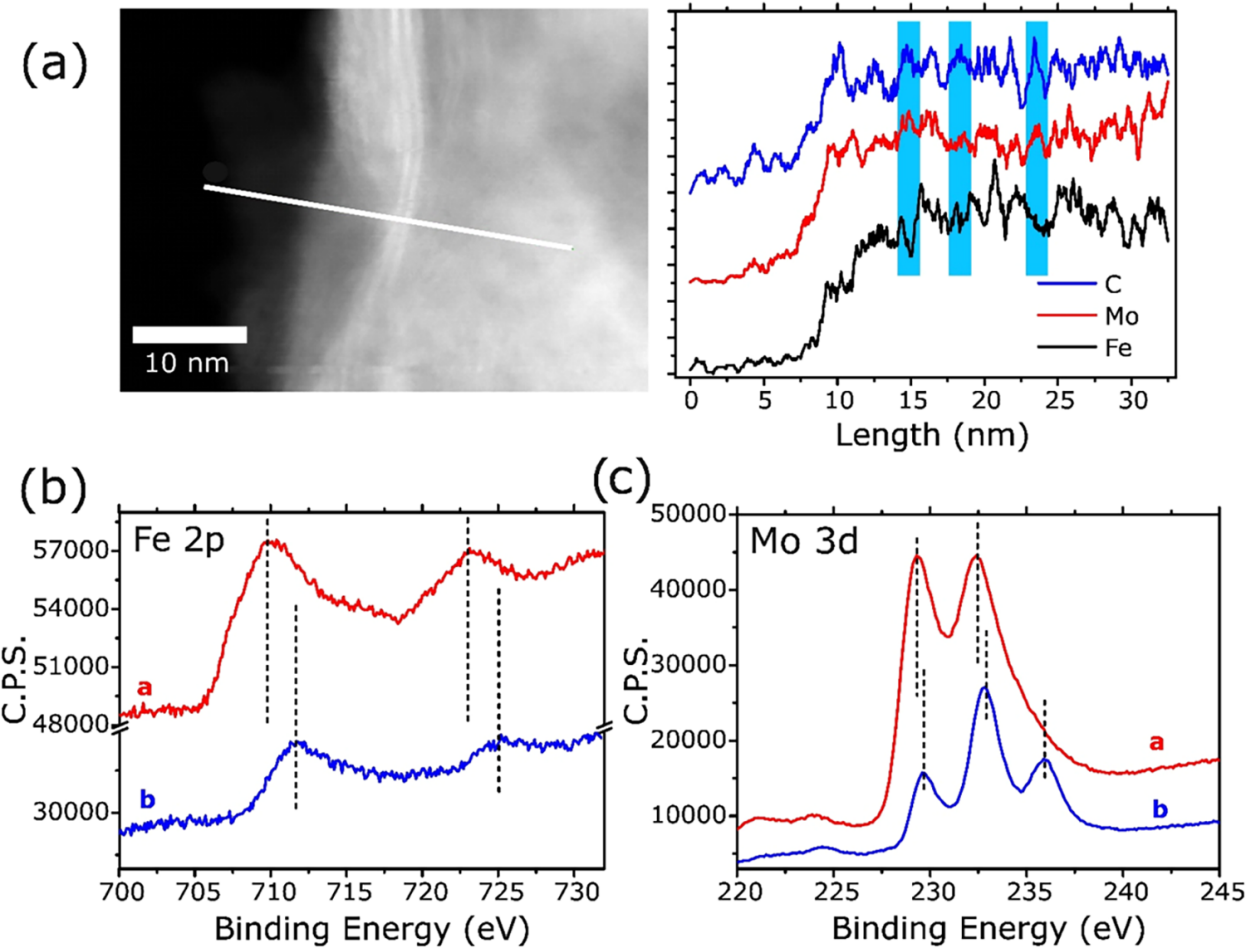
(a) STEM–EDX line scan across a Fe–Mo_2_C flake (left, ADF image; scale = 10 nm). Elemental profiles (right) show C (blue) and Mo (red) rising uniformly within the platelet, while Fe (black) appears only inside the particle and exhibits three periodic minima for C and Mo maxima (shaded), pinpointing metallic Fe confined to successive interlayer galleries. (b, c) Overlaid XPS signals of Fe 2p (left) and Mo 3d (right). The blue curves represent measurements taken from the untreated Fe/Mo_2_C-3 powder, reflecting the redox states within the top few nanometers of the surface. In contrast, the red curves correspond to spectra obtained after Ar^+^ sputtering removal of the superficial layers, revealing less oxidized redox states beneath the catalyst surface.

To probe the chemical stratification of the catalyst, we performed X-ray photoelectron spectroscopy (XPS) in two stages: (i) a surface spectrum collected under ultrahigh vacuum immediately after sample introduction (Figure S6), and (ii) a subsurface spectrum acquired after Ar^+^ sputtering at 5 kV and a pressure of 1 × 10^–7^ mbar for 20 min (estimated erosion ≈ 4–5 nm) (Figure S7). The depth profile reveals pronounced compositional and oxidation-state gradients.

In the XPS Mo 3d spectrum, the as-received surface shows a prominent doublet at 232.3 eV (3d_5/2_)/235.3 eV (3d_3/2_), diagnostic of Mo^6+^ (MoO_3_) formed by air oxidation of the topmost basal plane. After sputtering, this high-BE feature vanishes, and the spectrum collapses into the lower-energy doublets centered at 229.1 and 230.3 eV that we assign to Mo-oxy-carbide (Mo^3+^) and Mo^4+^, respectively. The result confirms that the MoO_3_ skin is no more than a few nanometers thick and that the bulk of the flake remains in the carbidic state.

The Fe 2p region undergoes an even more striking transformation. In the surface scan, only oxidized components are present (Fe^2+^ at ∼710.4 eV and Fe^3+^ at ∼712.3 eV). Post-sputtering, a new intense peak appears at 707.9 eV (2p_3/2_). This binding energy is characteristic of zerovalent iron or Fe-carbide and is completely absent in the unsputtered spectrum, implying that metallic Fe is buried beneath the oxidized surface layer. Its emergence only after removing ∼5 nm of material corroborates the structural model derived from HR-STEM, where Fe(0) is confined within the van der Waals galleries of Mo_2_C rather than decorating the external surface.

Figure 4b,c overlays the high-resolution XPS Fe 2p and Mo 3d spectra, respectively, acquired on the as-received surface (blue curves) with those recorded after Ar^+^ sputtering (orange curves). In both panels, the XPS peaks of the as-received samples are shifted to higher binding energies, evidencing more oxidized species –Mo^6+^ at 232.3 eV and Fe^2+^/Fe^3+^ at 710–713 eV, whereas the sputtered XPS peaks reveal the lower-BE doublets of carbidic Mo and metallic Fe/Fe-carbide (≈707.9 eV). The comparison confirms that the oxidized layer is confined to the outermost few nanometers and that zerovalent Fe is buried between the Mo_2_C sheets.

Full peak deconvolutions for both the as-received and sputtered XPS spectra, including individual component fits and detailed chemical assignments, are provided in Figures S6 and S7. An additional comment refers to the low Fe cross section and low electron escape efficiency for Fe in comparison to other metals.^25^ For these reasons, together with the distribution of Fe atoms among various oxidation states, quantitative analysis of Fe by XPS is subjected to a considerable error that precludes a direct comparison with the ICP analysis values that are far more accurate, although the results refer to the distribution of Fe in the bulk of the material.

Electron energy loss spectroscopy (EELS), performed in conjunction with HR-STEM, was employed to investigate the spatial distribution of iron and its oxidation states within the catalyst material. Line scans were conducted across selected regions, and spectra were acquired at three distinct positions: outside the material, at its edge, and within the central bulk. This approach enables a comparative analysis of iron signal intensity across different structural zones of the catalyst, as shown in Figure S8. The intensity of the iron peaks in the center of the catalyst particle is higher, leading to the conclusion that there is more iron than on the edge due to the particle being more in bulk and more layered being scanned. The intensity ratio *I*_L3_/*I*_L2_ at the interior of the Fe–Mo_2_C particle is 5.1 and at the surface *I*_L3_/*I*_L2_ is approximately 2. This ratio relates to the oxidation state, where lower ratios suggest a certain oxidation of the iron, whereas higher ratios indicate a more reduced valence state.^26–28^ With a high ratio of 5 on the edge of the material, the iron is in an oxidized state, whereas in the center of the catalyst particle, the iron is more reduced, which complements the XPS measurements, also showing a change of oxidation state depending on the penetration depth in the material.

The eNRR performances of the prepared materials were evaluated using a N_2_-saturated 0.5 M Na_2_SO_4_ solution under ambient conditions using an H-cell system. Figure S9 shows the LSV of all four materials in Ar- and N_2_-saturated 0.5 M Na_2_SO_4_ solution. As shown in Figure 5a, deposition of Fe over 5 wt % increases eNRR performance with respect to pristine Mo_2_C. Based on reported electrochemical impedance spectroscopy for Co–Fe deposition on Mo_2_C for HER and OER,^29,30^ the effect of metal deposition can be due to a diminished charge-transfer resistance and higher electrochemical surface area. Among the series, the Fe/Mo_2_C-3 sample exhibited the most favorable electrocatalytic performance, as evidenced by the most positive onset potential and a sharp increase in current density under N_2_, clearly deviating from the corresponding Ar counterpart. In contrast, the other samples displayed more negative onset potentials. These results point to Fe/Mo_2_C-3 as the most efficient catalyst. Figure S10 shows the LSV of the pristine Mo_2_C and the Fe/Mo_2_C-3 catalyst; the overpotential at 10 mA cm^–2^ was −0.60 V vs RHE for Fe/Mo_2_C-3 in N_2_, while the pristine Mo_2_C exhibited a higher overpotential of −0.79 V vs RHE under the same conditions. Additionally, the Tafel slope (Figure S11) of the bare Mo_2_C was significantly larger than that of the Fe/Mo_2_C-3, suggesting improved catalytic activity upon Fe impregnation.

**Figure 5:**
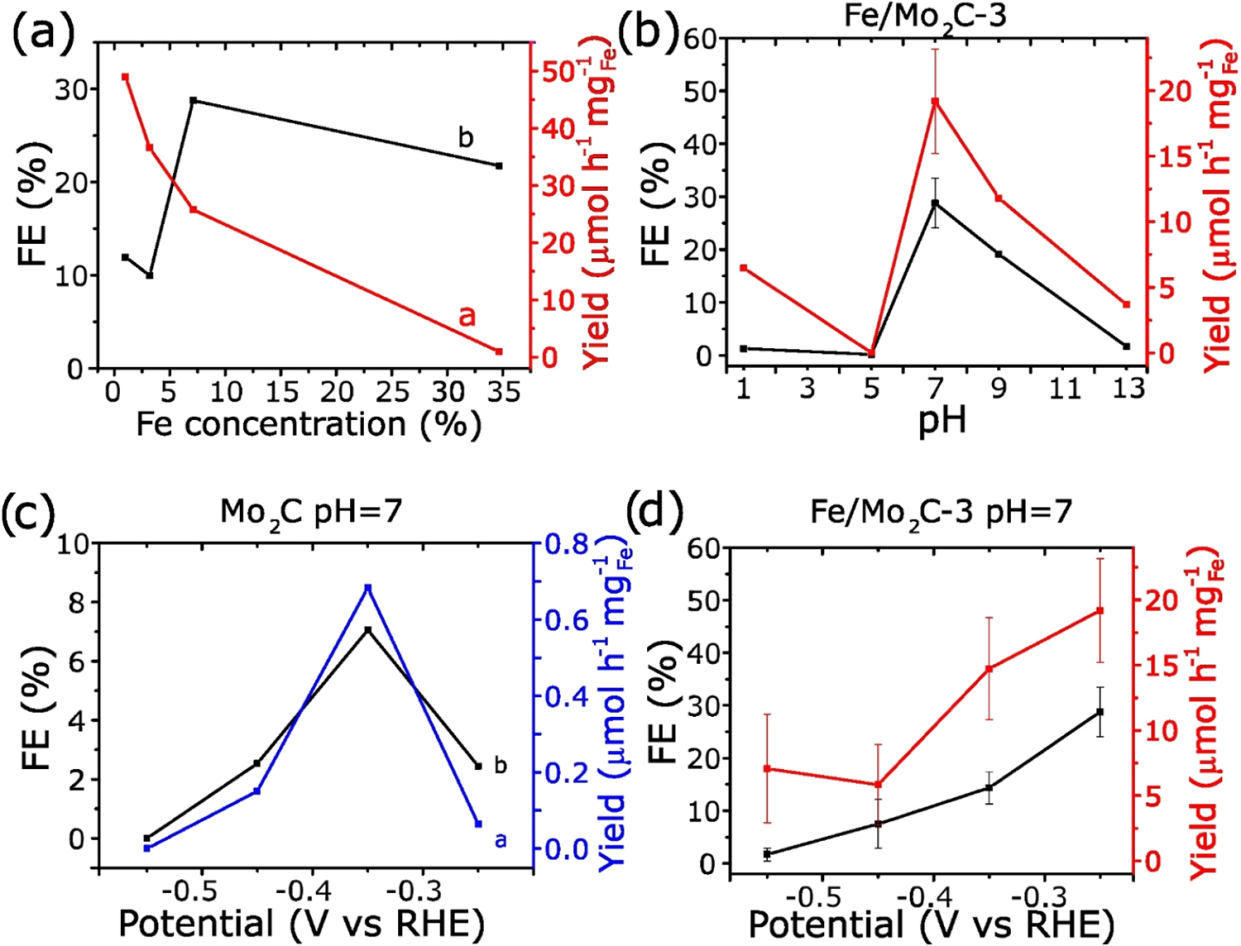
(a) Influence of iron concentration of the Fe/M_2_C series on the FE and on the catalyst-normalized yield, evaluated in 2 h CA experiments at −0.25 V vs RHE in neutral 0.1 M Na_2_SO_4_. (b) Analysis of the influence of the pH of the electrolyte on the FE and iron-normalized yield, measured with Fe/Mo_2_C-3 at −0.25 V vs RHE. Comparison of FE and normalized yield over several applied potentials for (c) pristine Mo_2_C MXene and (d) Fe/M_2_C-3. Electrolyte was 0.1 M Na_2_SO_4_.

Figure 5a and Table S4 display the influence of Fe concentration on the performance of all four Fe/Mo_2_C materials for the electrochemical NRR evaluated through 2 h CA measurements conducted at an applied potential of −0.25 V vs RHE using N_2_-saturated 0.5 M Na_2_SO_4_ solution. This potential was selected based on comparative CA measurements at −0.25 V vs RHE and −0.35 V vs RHE (Table S3), which showed that the best overall NRR performance (considering both FE and mass-normalized yield) was obtained at −0.25 V vs RHE. No correction for the ohmic drop and internal resistance of the H-cell was applied, and the experimental measured *V* and *J* values were used in the calculations as if the H-cell had no ohmic losses. Among the Fe/Mo_2_C series, Fe/Mo_2_C-3 (with 7% Fe content) emerged as the optimal catalyst, achieving the best balance between activity and selectivity. As shown in Table S4 and Figure 5a, Fe/Mo_2_C-3 delivers the highest FE (28.78%) and catalyst-normalized NH_3_ yield (1.34 μmol h^–1^ mg_cat_^–1^) in the series. While Fe/Mo_2_C-1 (with 1% Fe content) exhibits the highest Fe-normalized yield of 49.55 μmol h^–1^ mg_Fe_^–1^ in the series of samples, its significantly lower FE (12%) indicates poor selectivity and limited overall productivity. The results indicate that a content near 7% is optimal for maximizing eNRR performance under these conditions, highlighting Fe/Mo_2_C-3 as the most efficient catalyst and supporting the LSV observations.

Following the identification of Fe/Mo_2_C-3 as the most effective catalyst based on CA and LSV results, additional CA experiments were conducted to evaluate its performance under varying reaction environments. Specifically, Fe/Mo_2_C-3 was tested in five different electrolytes spanning a wide pH range from 1 to 13 and across an extended potential window from −0.25 V vs RHE to −0.55 V vs RHE (Figures S12 and 5d) to explore its activity and selectivity across different electrochemical conditions. The best performance was observed under initial neutral pH at an applied potential of −0.25 V vs RHE, achieving a FE of 28.78% and a yield of 19.14 μmol h^–1^ mg_Fe_^–1^ (1.34 μmol h^–1^ mg_cat_^–1^), Figure 5d, as previously described. Worth noting is that the pH value at the end of the measurement becomes basic. Under slightly initial basic conditions, the catalyst showed a somewhat lower yet still comparable performance with a FE of 19.1% and a yield of 11.78 μmol h^–1^ mg_Fe_^–1^ at the same potential (Figure S12). In contrast, under strongly acidic or strongly alkaline conditions (Figure S12), the FE dropped below 1%, and yields were lower than 6.5 μmol h^–1^ mg_Fe_^–1^, indicating poor NRR activity in these extreme pH environments.

There seems to be no consensus on the optimal pH for the NRR in aqueous electrolyte. At acidic pH, the parasitic HER becomes dominant due to a higher concentration of protons and thereby suppressing the NRR toward ammonia formation.^31^ Additionally, it has been reported that the oxidation state of the elements in Mo_2_C changes in acidic media, possibly changing the electronic state to a less favorable state. Looking into Mo_2_C in alkaline media, it has been reported that Mo_2_C MXene is unstable at higher pH, leading to a lower ammonia production rate after decomposition of the MXene.^32,33^ The reports indicating instability in alkaline media and dominance of the HER under acidic conditions further underscore why our catalyst exhibits optimal performance in neutral media.

These results are summarized in Figure 5b, which shows the pH-dependent performance of Fe/Mo_2_C-3 at −0.25 V vs RHE. For comparison, the pristine Mo_2_C was also tested at neutral pH (Figure 5c) across an extended potential window. It was found that Fe/Mo_2_C-3 outperforms pristine Mo_2_C in both efficiency and yield. Furthermore, to confirm that the measured ammonia was in fact produced by N_2_ feeding gas and it was not contamination, ^15^N_2_ isotopic labeling experiment was performed at neutral pH and an applied potential of −0.25 V vs RHE. The ^1^H NMR spectra (Figure S14) revealed a doublet proving the measured ammonia was in fact ^15^NH_4_^+^.^34,35^

Furthermore, the absence of a triplet accompanying this doublet conclusively rules out the contribution of any ^14^NH_4_^+^ that could be also present coming from other sources different from gaseous ^15^N_2_, like electrolyte, membrane, or the ambient atmosphere.

To benchmark the performance of our FeMo_2_C-3 catalyst, we compiled in a comparative table (Table S5) data from electrocatalysts reported in the literature with outstanding FE (exceeding 25%), focusing on MXene-based materials, iron-containing nanostructures, and iron single-atom catalysts (SACs). Fe/Mo_2_C-3 achieves a FE of 28.78% and an ammonia yield of 1.34 μmol h^–1^ mg_cat_^–1^ at −0.25 V in neutral 0.1 M Na_2_SO_4_, placing it among the top-performing MXene-based systems. For comparison, a single-atom Ru-doped Mo_2_CT_*x*_ Mxene exhibited a higher yield of 2.39 μmol h^–1^ mg_cat_^–1^ but a lower FE of 25.77% under similar conditions (−0.3 V, 0.5 M K_2_SO_4_),^24^ while relying on a scarce and expensive noble metal (Ru). When normalized by the iron content, Fe/Mo_2_C-3 achieves a yield of 19.14 μmol h^–1^ mg_Fe_^–1^, lower than that of some SACs, yet with a considerably higher Fe loading. Moreover, unlike many SACs that require alkaline conditions to achieve high efficiencies, Fe/Mo_2_C-3 performs effectively under neutral conditions. Compared to other Fe-based materials, Fe/Mo_2_C-3 matches or exceeds the performance of systems such as Fe-doped TiO_2_ nanoparticles, Mo–Fe carbide, and Fe_3_O_4_ nanosheets across various pH conditions.^36–38^ While some materials, like fluorine-doped Fe nanoparticles on graphene,^39^ have reported higher FE values (up to 41.6%), they typically operate in alkaline media and may involve more tedious fabrication methods. This balance of selectivity, activity, and environmentally benign operations underscores the promise of Fe-doped MXenes as practical and scalable electrocatalysts for NRR.

To investigate the reaction mechanism, in situ attenuated total reflection surface-enhanced infrared absorption spectroscopy (ATR-SEIRAS) was performed. Figure 6a presents the ATR-SEIRAS data recorded on the Fe/Mo_2_C-3 surface under various applied potentials in a N_2_-saturated electrolyte. The measurement was made over time, with the intermediates accumulating as the potential increases. Since intermediates accumulate, the change in the area under the peaks reflects how many intermediates are adsorbed on the catalyst surface at each potential, helping us identify the most efficient operating point for the catalyst. The peak at 1465 cm^–1^, which is indicative of an adsorbed amine group on the surface, grows over time/while increasing the potential. The area below the peak is proportional to the adsorbed species.^40^ We took the area of the peak at 1465 cm^–1^, calculated the derivative and plotted it in Figure S13 to see the change in adsorption of amine species on the surface. The biggest increase of surface coverage of amine species was from the potential of 0 to −100 mV vs RHE. The change in adsorbed species is lower at higher overpotential and stagnates around −400 mV vs RHE. It is assumed that at a potential of −300 mV vs RHE and higher, most of the readily available and electrochemical active surface area is already covered. This hypothesis also stands in agreement with our measured optimum of a Faradaic efficiency at −250 mV vs RHE. With the help of the ATR-SEIRAS, we can make out the types of intermediate species attached to the catalyst surface and therefore determine the pathway. The most prominent feature, observed at 3439 cm^–1^, is attributed to the O–H stretching vibration, which is indicative of adsorbed hydroxyl species. Adjacent to this, a subtle shoulder at 3218 cm^–1^ slowly emerging from −100 mV vs RHE on is assigned to the N–H stretching mode, commonly associated with ammonia or amine-like species. This implies the existence of the electrochemically adsorbed amine intermediate, indicating its role in the catalytic cycle starting at low overpotentials. A distinct band at 1647 cm^–1^ corresponds to N–H bending, while the peak at 1465 cm^–1^ is characteristic of H–N–H scissoring vibrations. Further, the band at 1241 cm^–1^ is attributed to –NH_2_ wagging, and the signal at 1114 cm^–1^ is assigned to N–N stretching.^41–44^ The simultaneous presence of –NH_2_ wagging and N–N stretching vibrations strongly supports the involvement of an associative pathway in the nitrogen reduction mechanism in which successive e^–^ and H^+^ are incorporated before the N≡N bond is broken.

**Figure 6:**
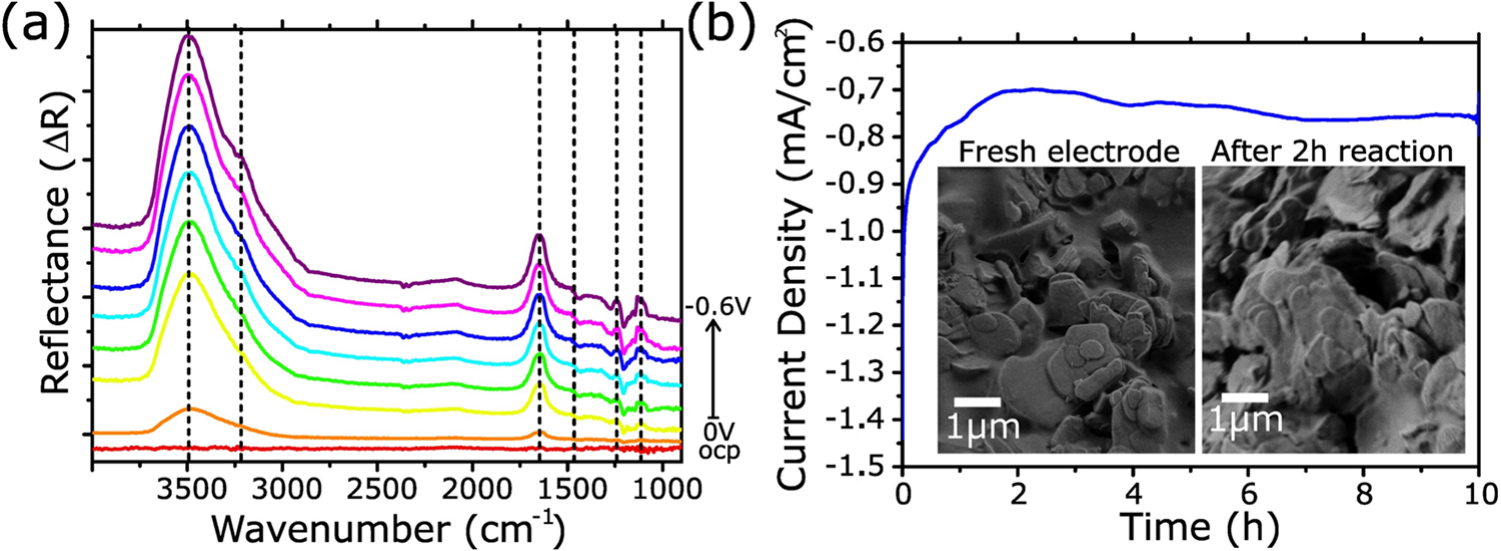
(a) ATR-SEIRAS spectra for Fe/Mo2C-3 collected at various applied potentials from open circuit potential (ocp) to −600 mV vs RHE, showing the evolution of vibrational features associated with adsorbed ammonia intermediates. (b) Stability test: CA at a potential of −0.25 V vs RHE for 10 h with Fe/Mo2C-3 at neutral pH. Inset showing SEM images of the electrode before and after 2 h reaction at −0.25 V vs RHE in neutral electrolyte.

The stability of Fe/Mo_2_C-3 was evaluated through a long-term CA test of 10 h conducted at −0.25 V vs RHE in neutral media. As shown in Figure 6b, the current remained stable throughout the experiment, with only a slight increase of 0.05 mA/cm^2^ in current density, indicating good electrochemical durability under NRR conditions. Furthermore, SEM images (Figure 6b, inset) before and after the long-term CA experiments suggest that the morphology of the material is maintained. To assess possible iron leaching, postreaction analysis of the electrolyte was performed using ICP-OES. Interestingly, no iron was detected in the electrolyte (−0.007 ppm Fe by ICP, indicating a value below the detection limit), despite Fe/Mo_2_C-3 containing 7% iron. The absence of Fe leaching suggests that iron-decorated regions of the MXene exhibit enhanced structural and chemical stabilities compared to bare MXene domains. This may be attributed to a physical stabilization through interlayer bridging by iron species, which reduces mechanical degradation from gas bubbling and electrolyte exposure.^45^ Additionally, during NRR, the iron sites likely act as the primary nitrogen adsorption centers, shielding the thinner Mo_2_C layers from direct chemical stress. These results support a synergistic interaction between Fe and Mo, combining the catalytic activity of Fe with the conductive support of the Mo_2_C matrix.

Impedance spectroscopy measurements were conducted at low frequencies, observing the characteristic half-circle. At lower frequencies, a spiral-like response emerged, attributed to the influence of adsorbed species on the surface (Figure S15). This phenomenon suggests enhanced conductivity and reduced resistivity due to the dynamic adsorption and desorption of species, which facilitate charge exchange with the surface. The inductive loop only appears when the adsorption and desorption rates are balanced and charge exchange occurs within the adsorbed/desorbed species, leading to the observed decrease in resistivity. Additionally, the inductive loop manifests only at a specific potential, where the adsorption rate is nearly equal to the desorption rate. The semicircle intersects the real axis at 20 Ω, corresponding to the solution resistance. The diameter of the semicircle is estimated to be around 1950 Ω. This behavior suggests a well-defined electrochemical process with minimal diffusion limitations.^46^

## Conclusions

In summary, the two-step strategy—in situ HF etching of Mo_2_Ga_2_C followed by Fe^2+^ impregnation and mild reduction—yields a conformal Fe overcoat on Mo_2_C MXene in which zerovalent iron forms an epitaxial monolayer on the basal planes while simultaneously occupying the intergallery voids of the 2D carbide, thereby reinforcing the lamellar architecture and providing continuous electron pathways without compromising crystallinity. High-resolution STEM/EDX and depth-resolved XPS confirm the metallic Fe on the Mo_2_C scaffold. Electrochemical screening shows that the Fe/Mo_2_C-3 composition containing 7 wt % iron delivers the best overall nitrogen reduction performance, achieving a Faradaic efficiency of 28.8% and an NH_3_ yield of 1.34 μmol h^–1^ mg_cat_^–1^ at −0.25 V vs RHE in neutral electrolyte. Impedance spectroscopy attributes this activity to a lowered charge-transfer resistance and favorable adsorption dynamics. The catalyst operates stably for 10 h with no detectable Fe leaching, while ^15^N_2_ isotopic labeling confirms that ammonia originates exclusively from nitrogen reduction. Complementary operando ATR-SEIRAS identifies N–N and −NH_*x*_ vibrations consistent with an associative pathway centered on Fe sites. Therefore, the combined structural and electrochemical evidence firmly establishes Fe-intercalated Mo_2_C as an earth-abundant electrocatalyst for ambient ammonia electrosynthesis.

## Supplementary Material



## Notes

Experimental methods; ICP-OES analyses; calibration plots for ammonia determination; total Fe in the impregnation solution vs Fe deposited on the MXene; HR-STEM images and FFT analyses; EDX elemental mapping; XPS analyses; electrochemical data and results, LSVs, Tafel plots, and CAs; comparative table with benchmark catalysts; and ^1^H NMR spectra of ammonia samples of different isotopes. (PDF)
