# Supplementary material for: Epitaxial Fe Coating on Single- and Few-Layer Mo2C MXene as Highly Efficient Catalyst for Ambient Electrochemical Ammonia Synthesis
Source: ACS Appl Energy Mater. 2025 Sep 16;8(19):14542–53. doi: 10.1021/acsaem.5c02197 (PMC12522097; doi:10.1021/acsaem.5c02197)
Supplement: Supplementary file 1 [file ae5c02197_si_001.pdf]

Supporting Information

Epitaxial Fe Coating on Single and Few-Layer Mo_2C MXene as Highly Efficient Catalyst for Ambient Electrochemical Ammonia Synthesis

Sabine Eliane Midré, Liang Tian, Hermenegildo García*, Sara Goberna-Ferrón* and Ana Primo*

Instituto de Tecnología Química, Consejo Superior de Investigaciones Científicas-Universitat Politècnica de Valencia, Universitat Politècnica de Valencia, Av. De los Naranjos s/n, 46022 Valencia, Spain.

Hermenegildo García; hgarcia@itq.upv.es

Ana Primo; aprimoar@itq.upv.es

Sara Goberna-Ferrón; sgobfer@itq.upv.es

	Fe/Mo_2C-1	Fe/Mo_2C-2	Fe/Mo_2C-3	Fe/Mo_2C-4
**C (mol/l)**	0.001	0.01	0.015	0.1
**Mass $Fe(C_2H_3O_2)_2$ (g)**	0.0035	0.0348	0.052	0.348
**Fe wt% theoretical**	1%	10%	14%	53%
**Fe wt% ICP OES**	1%	3.2%	7.1%	34.7%

Table S1 Comparison between amount of Iron in the Impregnation solution (maximum theoretical that could be adsorbed by the MXene) and actually adsorbed amount (ICP-OES)

pH	Electrolyte / Buffer
**1**	0.1 M HCl
**5**	0.1 M Acetate Buffer
**7**	0.1 M Na_2SO_4
**9**	0.1 M Carbonate Buffer
**13**	0.1 M KOH

Table S2 List of Electrolytes used at different pH

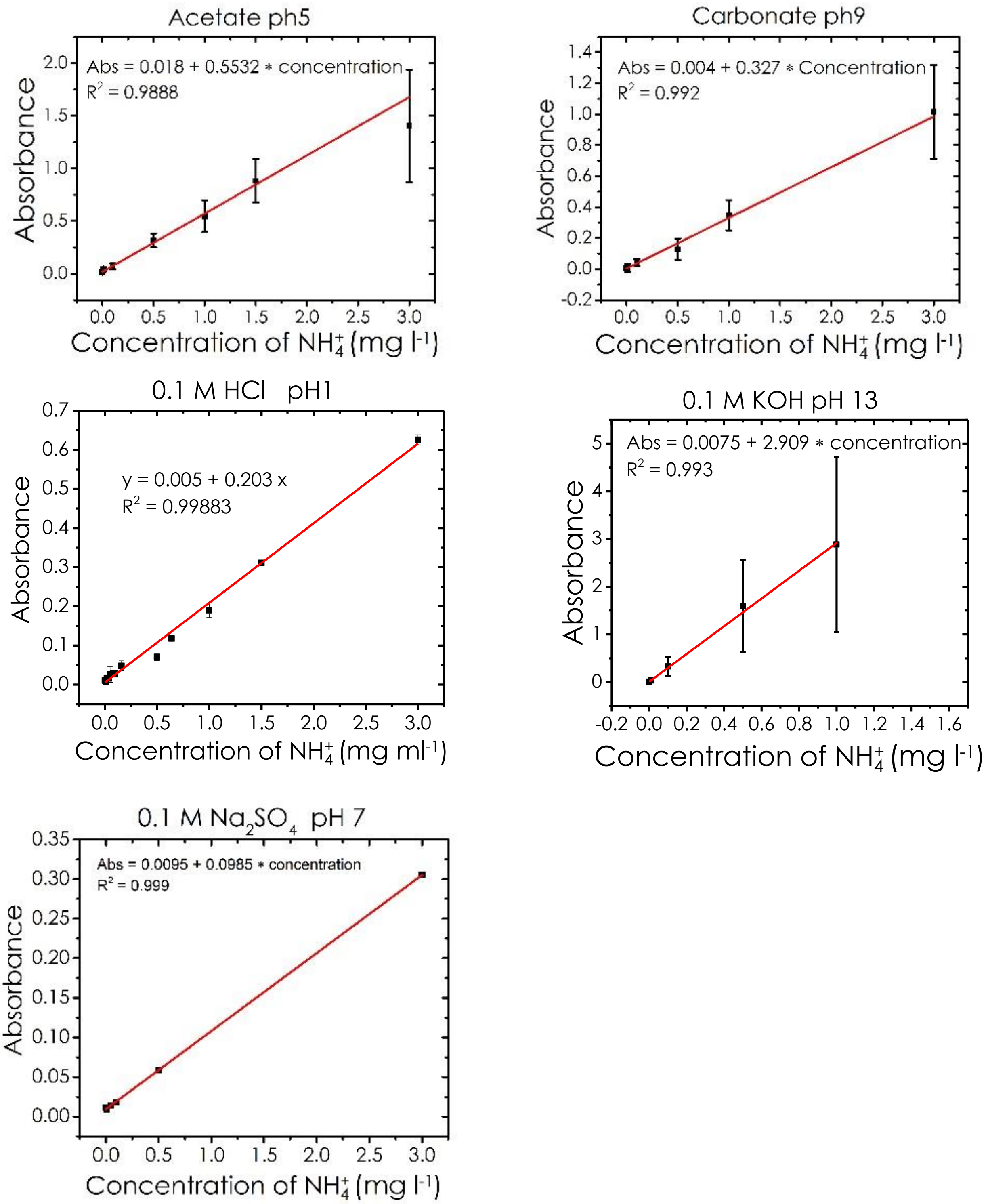

Figure S1 Calibration plots correlating absorbance of the indophenol kit and ammonia concentration in varying electrolytes and the R^2 Values indicating the reliability of the calibration.

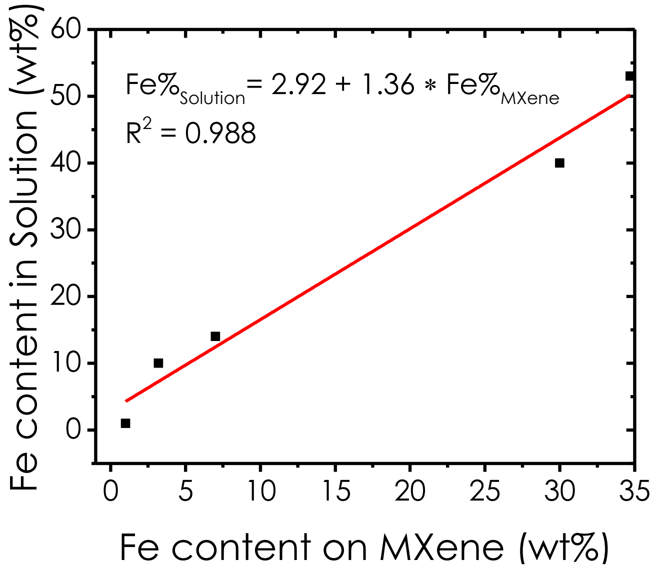

Figure S2 Linear relationship between total Iron Concentration in the impregnation Solution and Iron deposited on the MXene.

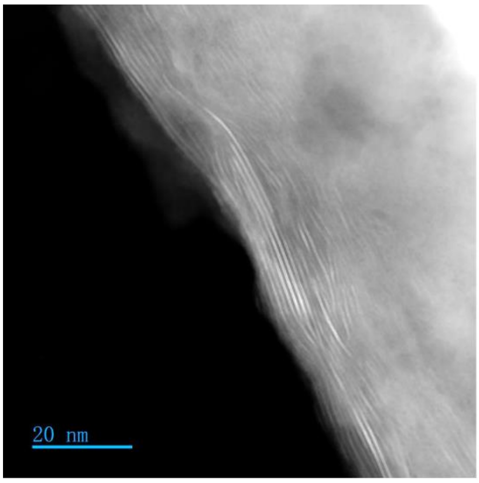

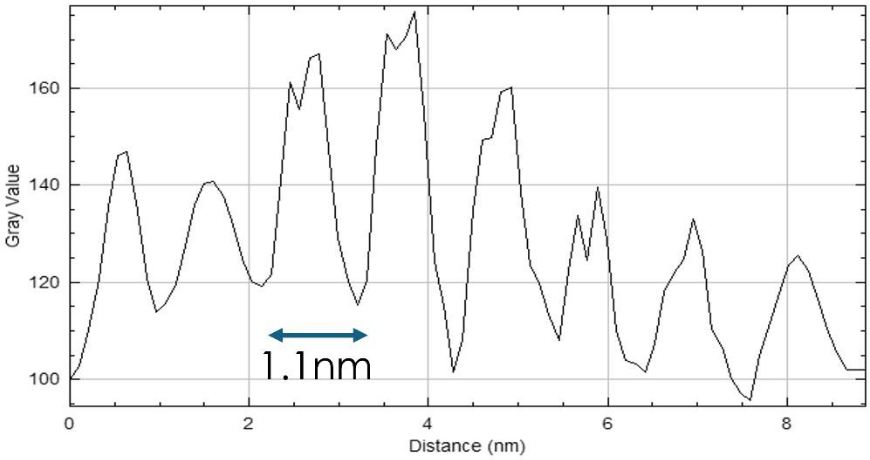

Figure S3 HR-STEM image of Fe/Mo_2C-3, showing layers from the side, with the profile plotted. The interlayer distance is shown to be 1.1 nm.

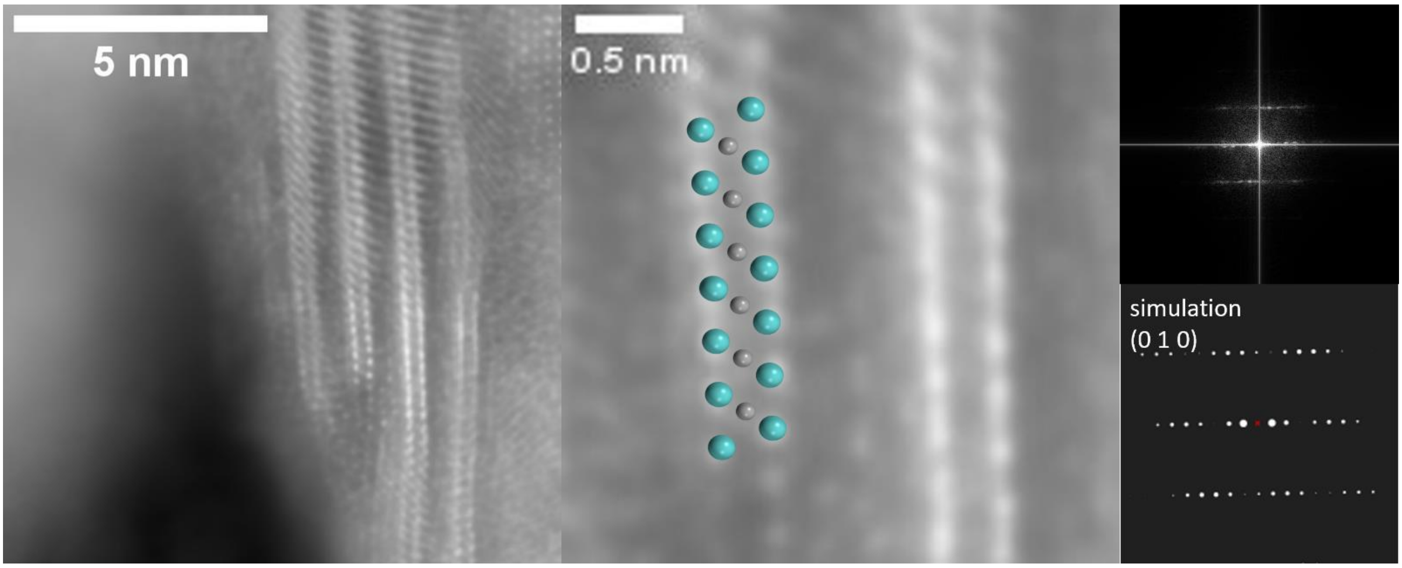

Figure S4 HR-STEM analysis of Fe/Mo_2C-3. The zoom shows the perfect fit of the image with the (0 1 0) facet. In this projection the Mo columns are aligned directly opposite one another across the interlayer, leading to the paired-dot motif seen experimentally. FFT of the excerpt fits the simulation of Mo_2C made with the software Recipro.

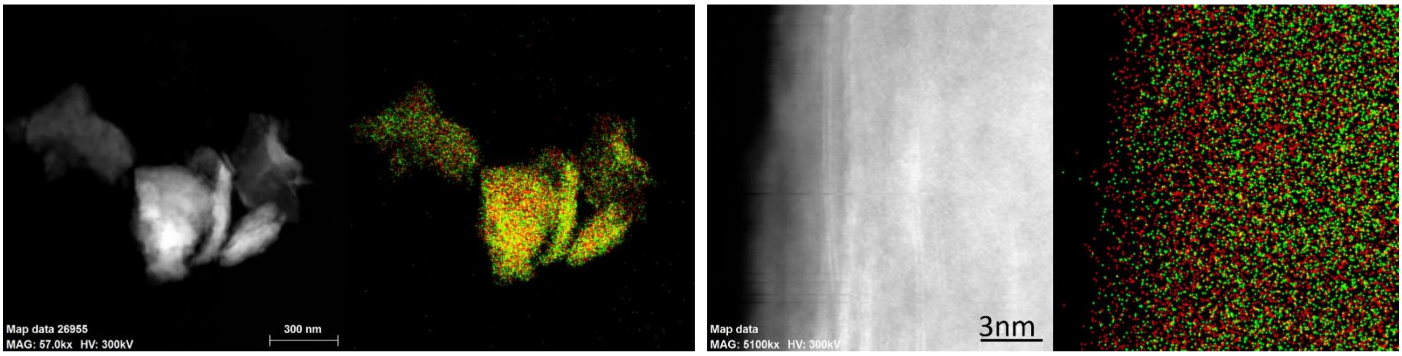

Figure S5 EDX measurement of Fe/Mo_2C-3 at different magnifications reveal well dispersed Iron over the MXene. Green signals are Iron and red signals are Molybdenum.

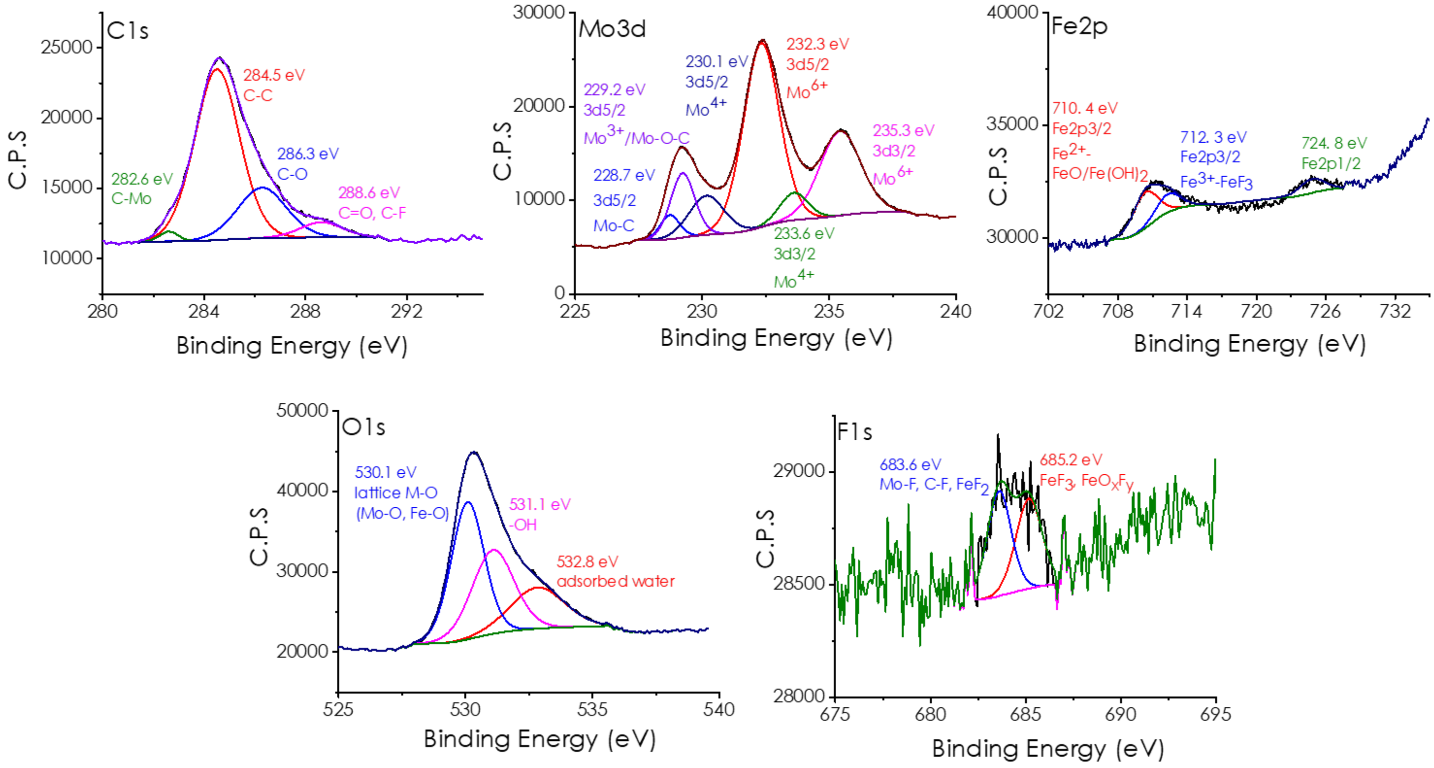

Figure S6. XPS peaks of the catalyst surface collected under ultrahigh vacuum with peak deconvolutions for C 1s, Mo 3d, Fe 2p, O 1s and F 1s. Colour-coded components identify carbidic Mo–C, graphitic/oxygenated carbon, $Mo^{2+/3+/4+/6+}$ species, Fe^{2+}/Fe^{3+} oxides, lattice/-OH oxygen and –F/FeF_x terminations.

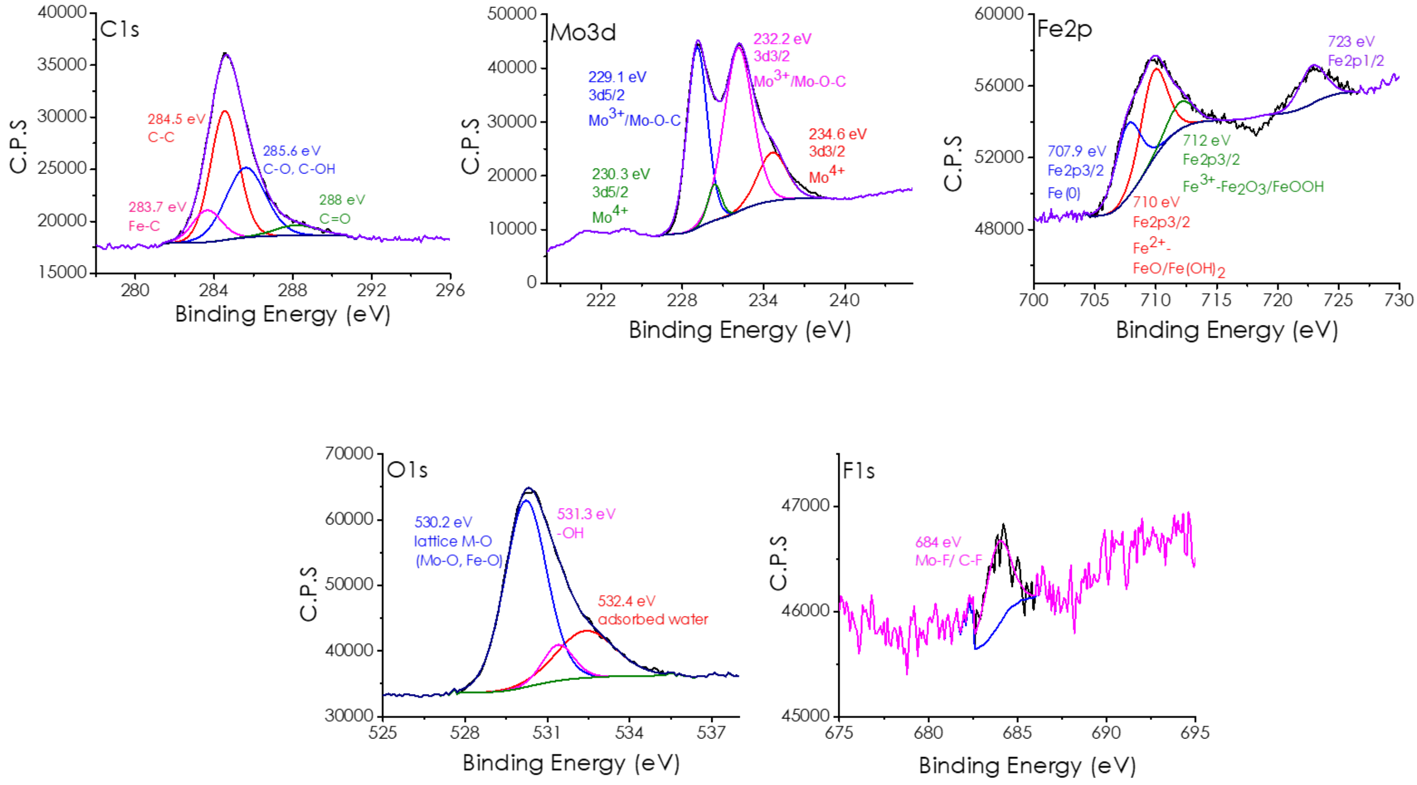

Figure S7. XPS data after Ar^+ sputtering (3 kV, 20 min) showing deconvoluted C 1s, Mo 3d, Fe 2p, O 1s and F 1s regions. Peaks reveal carbidic Mo–C and Fe^0/Fe-carbide, sub-surface Mo^{3+}/Mo^{4+} species, lattice/-OH oxygen and residual –F terminations, confirming the reduced, interlayer chemistry beneath the oxidised surface.

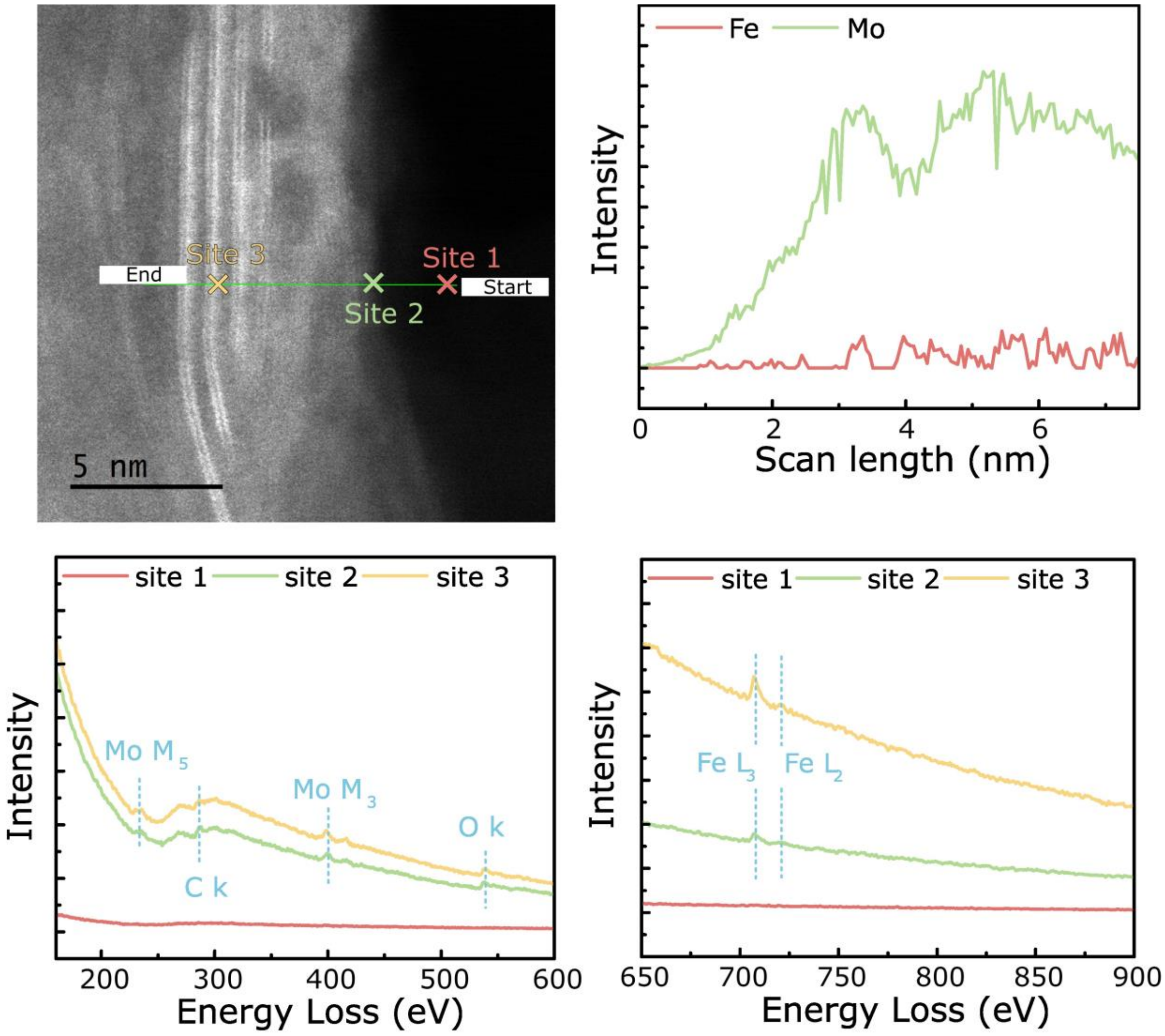

Figure S8. HRSEM-EELS data line scan, and the respective intensity of the Fe and Mo signals, getting stronger towards the inside of the particle. EELS deconvolution at 3 different sites, outside (blank), at the edge and in the center of the particle.

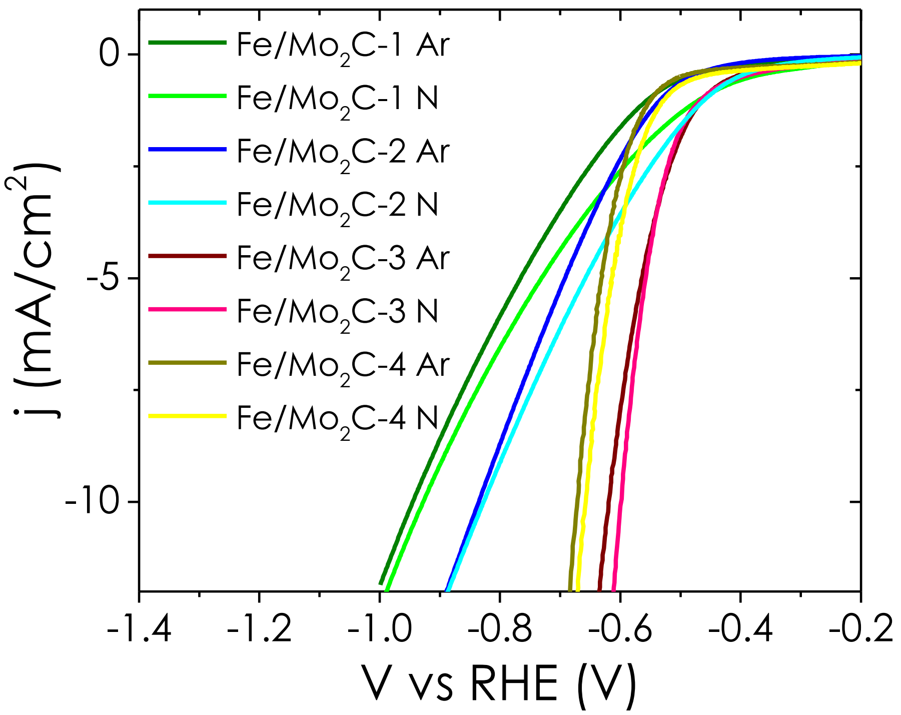

Figure S9 LSV comparison of Fe/Mo_2C-1, Fe/Mo_2C-2, Fe/Mo_2C-3 and Fe/Mo_2C-4, measured in Argon and Nitrogen Saturated Electrolyte. Electrolyte is 0.1 M Na_2SO_4.

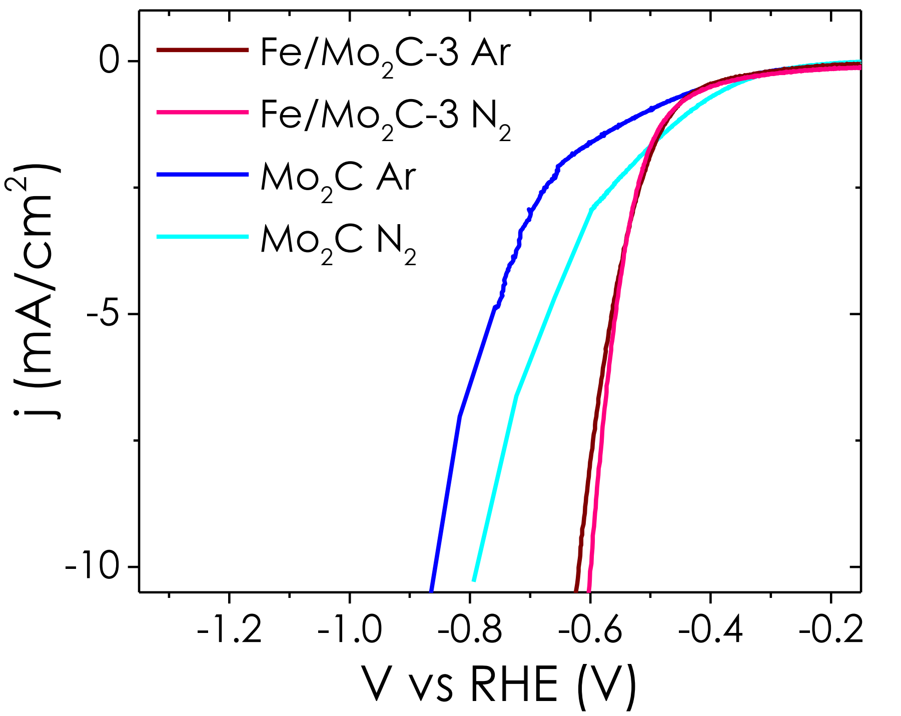

Figure S10 LSV comparison of pristine Mo_2C Mxene and Fe/Mo_2C-3, measured in Argon and Nitrogen Saturated Electrolyte. Electrolyte is 0.1 M Na_2SO_4. Fe/Mo_2C-3 shows the lowest overpotential.

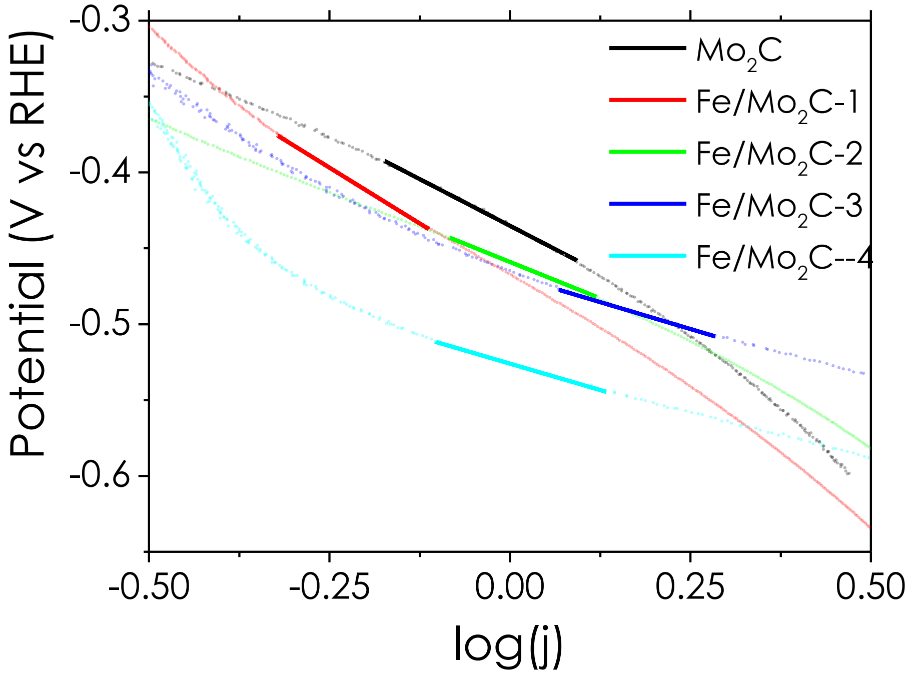

Figure S11 Tafel Plot and the respective Tafel Slopes of pristine Mo_2C MXene, Fe/Mo_2C-1, Fe/Mo_2C-2, Fe/Mo_2C-3 and Fe/Mo_2C-4. Measured in Nitrogen saturated Electrolyte, Electrolyte is 0.1M Na_2SO_4

	-0.25 V vs RHE		-0.35 V vs RHE	
	**FE (%)**	**Yield (µmol h^{-1} mg^{-1}cat)**	**FE (%)**	**Yield (µmol h^{-1} mg^{-1}cat)**
**MXene**	2.44	0.0645	7.06	0.68
**Fe/Mo_2C-1**	11.95	0.49	9.66	0.77
**Fe/Mo_2C-2**	9.99	1.17	10.54	1.43
**Fe/Mo_2C-3**	28.78	1.34	14.82	1.54
**Fe/Mo_2C-4**	21.75	0.32	19.67	1.35

Table S3. Results of a typical reaction, which consisted of 2h CA, followed by integration of the current and measurement of the ammonia yield. This leads to the calculation of the FEat the potentials -0.25V and -0.35V for screening purposes.

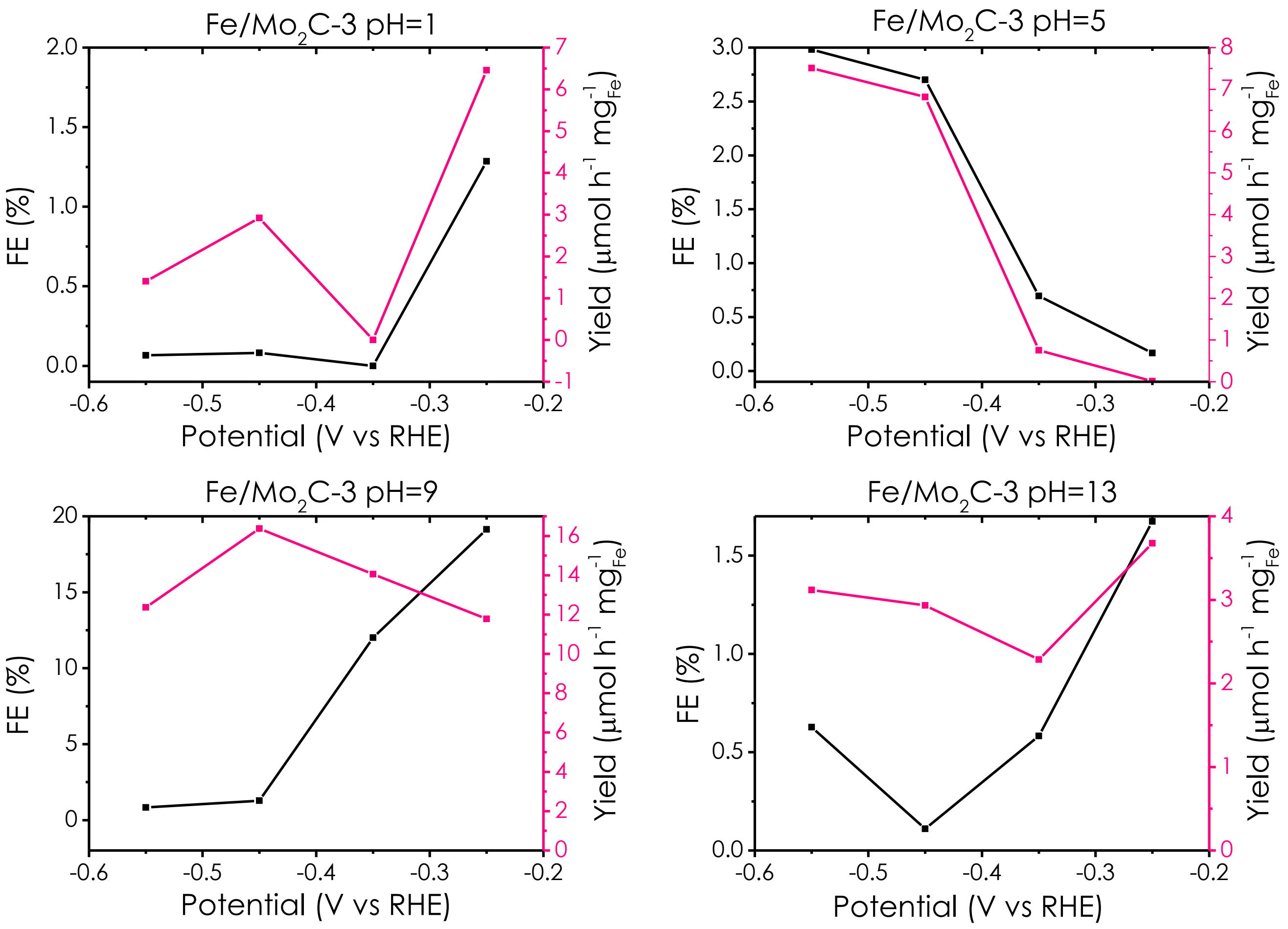

Figure S12 Performance of Fe/Mo_2C-3 in different Electrolytes at different pH

	MXene	Fe/Mo₂C-1	Fe/Mo₂C-2	Fe/Mo₂C-3	FeMo₂C-4
**FE %**	2.44	11.9	9.99	28.78	5.26
**Yield µmol h^{-1} mg_{Fe}^{-1}**	-	49.55	36.53	19.14	0.93
**Yield µmol h^{-1} mg_{cat}^{-1}**	0.06	0.49	1.17	1.34	0.32

Table S4. Yield normalized by amount of Iron, at 0.25 V vs RHE in neutral electrolyte

Catalyst	Electrolyte	NH_3 yield (μmol h^{-1} mg_{cat}^{-1})	NH_3 yield (μmol h^{-1} mg_{metal}^{-1})	FE (%)	Potential (V_{RHE})	Ref
**MXenes and MXene-based nanocomposites**						
**FeMxene-3 (this paper)**	**Neutral 0.1 M Na_2SO_4**	**1.34**	**19.14**	**28.78**	**-0.25**	
Single-atom Ru (1.41 %) / Mo_2CTx MXene	Neutral 0.5 M K_2SO_4	2.39	169.25	25.77	-0.3	[1]
MXene/TiFeOx (NPs, 10%Fe)	Acid 0.05 M H_2SO_4	1.29	12.88	25.44	-0.2	[2]
Bi NPs@Ti_3C_2 Mxene	Basic 0.1 M KOH	28.3 μg $h^{-1}cm^{-2}$		27.2	-0.4	[3]
**Fe-based electrocatalysts**						
Fe-doped TiO2 NPs	Neutral 0.5 M $LiClO_5$	1.50		25.6	-0.4	[4]
FeNPs on graphene doped with F	Basic 1M KOH	3.14		41.6	-0.38	[5]
Mo–Fe carbide	Neutral 0.1 M Li_2SO_4	0.44		27	-0.05	[6]
Fe_3O_4 nanosheets	Neutral 0.1 M Na_2SO_4	0.71		34.38	-0.1	[7]
4%Zr-doped α-FeOOH	Neutral 0.1 M Na_2SO_4	$1.39x10^{-10}$ mol/s cm^{-2}		35.63	-0.5	[8]
**Fe-based SACs**						
Fe_{SA} (1.09 %) / N-doped carbon	Basic 0.1 M KOH	0.44	40.47	56.55	0	[9]
Fe_{SA} (0.6 %) / graphitic C	Basic 0.1 M KOH	1.89	314.71	29.3	-0.1	[10]
Fe_{SA} (0.36 %) /N-doped carbon	Basic 0.1 M KOH	3.12	867.97	39.6	-0.35	[11]
Fe_{SA} /TiO_2 NPs	Acid 0.05 M H2SO4	1.64		27.67	-0.5	[12]

Table S5. Comparison of $FeMo_2C$-3 with other electrocatalysts for the NRR. The comparison fuses on catalysts with FE exceeding 25%.

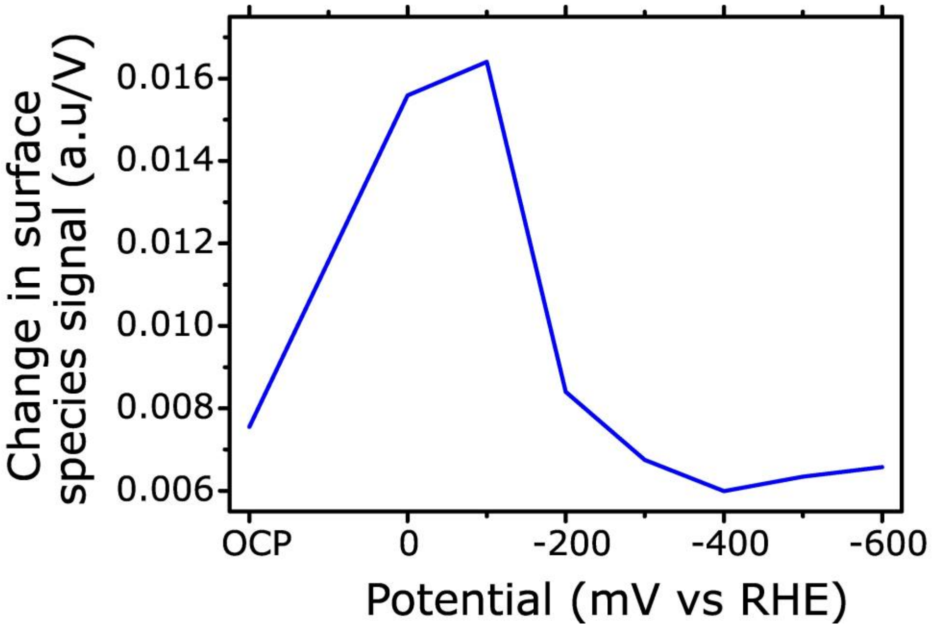

Figure S13 Derivative of ATR-SEIRAS signal showing the change in adsorbed amine species while lowering the potential over time

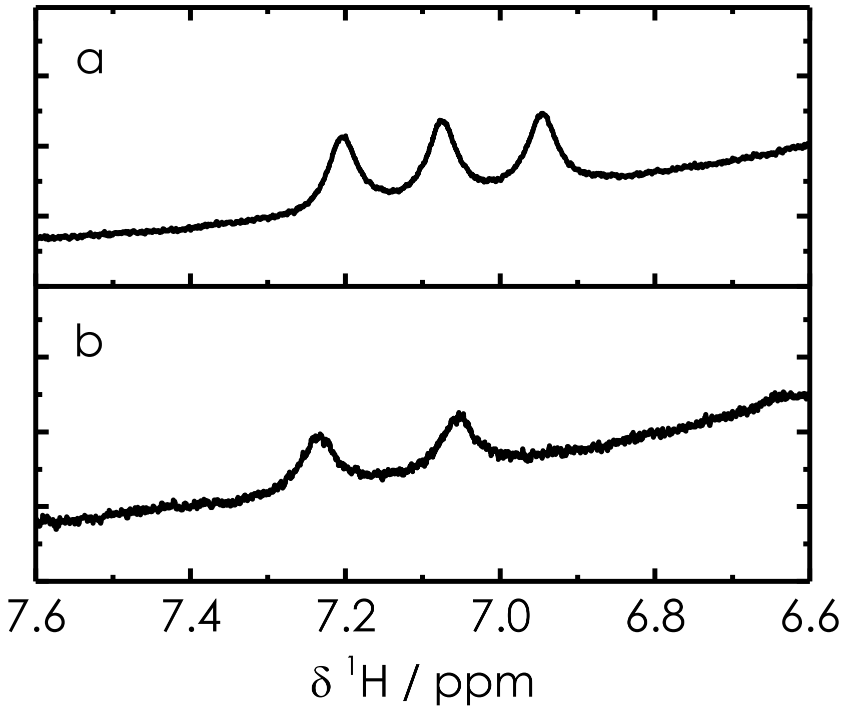

Figure S14. ^{1}H NMR spectra of ammonia samples of different isotopes. (a) Spectrum of commercial ammonia dissolved in 0.1 M Na_2SO_4 electrolyte, measured as a reference for comparison with isotopically labelled ammonia. The spectrum displays the characteristic triplet pattern of $^{14}NH_4^+$, arising from scalar coupling between the four equivalent protons and the quadrupolar ^{14}N nucleus (spin = 1), which has three magnetic sublevels. (b) Spectrum of ammonia collected from the working electrode compartment of an H-cell after a 2-hour electrochemical reaction under continuous $^{15}N_2$ gas purging at a bias of -0.25 V vs RHE at neutral pH. The resulting spectrum shows the expected doublet for $^{15}NH_3$, due to coupling between the four equivalent protons and the ^{15}N nucleus (spin = ½), which has two magnetic sublevels.

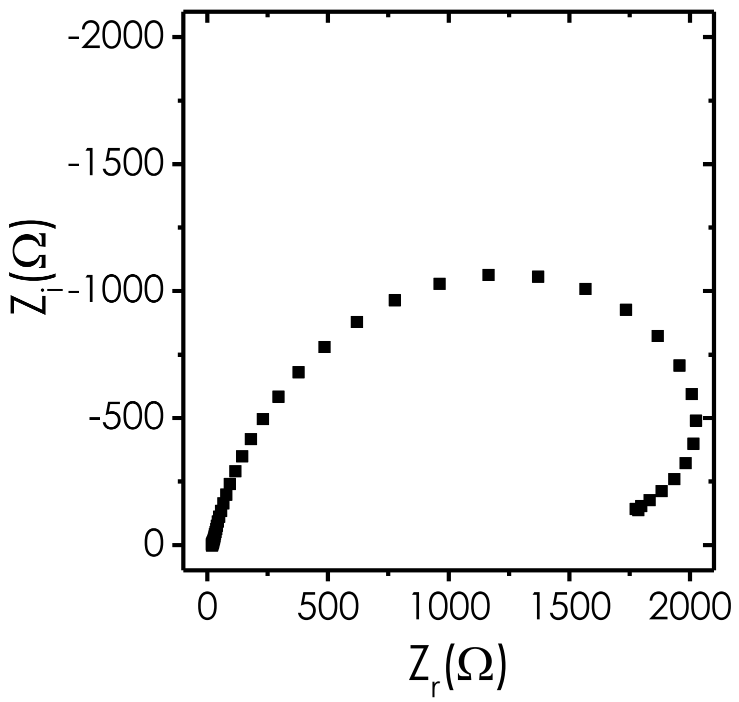

Figure S15. EIS measurement of Fe/Mo_2C -3s2 conducted at low frequencies, observing the characteristic half-circle

References

[1] W. Peng *et al.*, 'Spontaneous Atomic Ruthenium Doping in Mo2CTX MXene Defects Enhances Electrocatalytic Activity for the Nitrogen Reduction Reaction', *Adv Energy Mater*, vol. 10, no. 25, Jul. 2020, doi: 10.1002/aenm.202001364.

[2] Y. Guo *et al.*, 'Highly Efficient Electrochemical Reduction of Nitrogen to Ammonia on Surface Termination Modified Ti3C2TxMXene Nanosheets', *ACS Nano*, vol. 14, no. 7, pp. 9089–9097, Jul. 2020, doi: 10.1021/acsnano.0c04284.

[3] A. Liu *et al.*, 'Two-Dimensional MXene Supported Bismuth for Efficient Electrocatalytic Nitrogen Reduction', *ChemCatChem*, vol. 14, no. 7, Apr. 2022, doi: 10.1002/cctc.202101683.

[4] T. Wu *et al.*, ' Greatly Improving Electrochemical N 2 Reduction over TiO 2 Nanoparticles by Iron Doping ', *Angewandte Chemie*, vol. 131, no. 51, pp. 18620–18624, Dec. 2019, doi: 10.1002/ange.201911153.

[5] X. Wang *et al.*, 'Regulating the Electronic Configuration of Supported Iron Nanoparticles for Electrochemical Catalytic Nitrogen Fixation', *Adv Funct Mater*, vol. 32, no. 21, May 2022, doi: 10.1002/adfm.202111733.

[6] B. Qin, Y. Li, Q. Zhang, G. Yang, H. Liang, and F. Peng, 'Understanding of nitrogen fixation electro catalyzed by molybdenum–iron carbide through the experiment and theory', *Nano Energy*, vol. 68, Feb. 2020, doi: 10.1016/j.nanoen.2019.104374.

[7] H. Ying, T. Chen, C. Zhang, J. Bi, Z. Li, and J. Hao, 'Regeneration of porous Fe3O4 nanosheets from deep eutectic solvent for high-performance electrocatalytic nitrogen reduction', *J Colloid Interface Sci*, vol. 602, pp. 64–72, Nov. 2021, doi: 10.1016/j.jcis.2021.05.185.

[8] J. Tan, X. He, F. Yin, X. Liang, G. Li, and Z. Li, 'Zr-doped α-FeOOH with high faradaic efficiency for electrochemical nitrogen reduction reaction', *Appl Surf Sci*, vol. 567, Nov. 2021, doi: 10.1016/j.apsusc.2021.150801.

[9] M. Wang *et al.*, 'Over 56.55% Faradaic efficiency of ambient ammonia synthesis enabled by positively shifting the reaction potential', *Nat Commun*, vol. 10, no. 1, Dec. 2019, doi: 10.1038/s41467-018-08120-x.

[10] S. Zhang *et al.*, 'Electrocatalytically Active Fe-(O-C2)4 Single-Atom Sites for Efficient Reduction of Nitrogen to Ammonia', *Angewandte Chemie - International Edition*, vol. 59, no. 32, pp. 13423–13429, Aug. 2020, doi: 10.1002/anie.202005930.

[11] H. Yang, Y. Liu, Y. Luo, S. Lu, B. Su, and J. Ma, 'Achieving High Activity and Selectivity of Nitrogen Reduction via Fe-N3Coordination on Iron Single-Atom Electrocatalysts at Ambient Conditions', *ACS Sustain Chem Eng*, vol. 8, no. 34, pp. 12809–12816, Aug. 2020, doi: 10.1021/acssuschemeng.0c02701.

[12] G. Song *et al.*, 'High-spin state Fe(III) doped TiO2 for electrocatalytic nitrogen fixation induced by surface F modification', *Appl Catal B*, vol. 301, Feb. 2022, doi: 10.1016/j.apcatb.2021.120809.
